# FurA contributes to the oxidative stress response regulation of *Mycobacterium avium* ssp. *paratuberculosis*

**DOI:** 10.3389/fmicb.2015.00016

**Published:** 2015-02-06

**Authors:** Elke Eckelt, Thorsten Meißner, Jochen Meens, Kristin Laarmann, Andreas Nerlich, Michael Jarek, Siegfried Weiss, Gerald-F. Gerlach, Ralph Goethe

**Affiliations:** ^1^Institute for Microbiology, Department of Infectious Diseases, University of Veterinary Medicine HannoverHannover, Germany; ^2^Genome Analytics, Helmholtz Centre for Infection ResearchBraunschweig, Germany; ^3^Molecular Immunology, Helmholtz Centre for Infection ResearchBraunschweig, Germany

**Keywords:** oxidative stress response regulation, *fur*A, mycobacteria, iron, metallo-regulator

## Abstract

The ferric uptake regulator A (FurA) is known to be involved in iron homeostasis and stress response in many bacteria. In mycobacteria the precise role of FurA is still unclear. In the presented study, we addressed the functional role of FurA in the ruminant pathogen *Mycobacterium avium* ssp. *paratuberculosis* (MAP) by construction of a *fur*A deletion strain (MAPΔ*fur*A). RNA deep sequencing revealed that the FurA regulon consists of repressed and activated genes associated to stress response or intracellular survival. Not a single gene related to metal homeostasis was affected by *fur*A deletion. A decisive role of FurA during intracellular survival in macrophages was shown by significantly enhanced survival of MAPΔ*fur*A compared to the wildtype, indicating that a principal task of mycobacterial FurA is oxidative stress response regulation in macrophages. This resistance was not associated with altered survival of mice after long term infection with MAP. Our results demonstrate for the first time, that mycobacterial FurA is not involved in the regulation of iron homeostasis. However, they provide strong evidence that FurA contributes to intracellular survival as an oxidative stress sensing regulator.

## INTRODUCTION

For efficient infection and colonization, pathogenic bacteria need to adapt their metabolism to the host environment and to combat innate antimicrobial host defense mechanisms such as iron limitation by iron sequestering molecules such as ferritin, transferrin and lactoferrin, and the production of toxic reactive oxygen (ROS) and nitrogen species (RNS).

Reactive oxygen and nitrogen species comprise highly reactive molecules such as the superoxide anion (O_2_^-^), hydroxyl radical (OH^-^), and peroxynitrite (ONOO^-^) as well as more stable oxidants like hydrogen peroxide (H_2_O_2_) and nitric oxide (NO). These products are generated during auto oxidative metabolic processes and play important roles in prokaryotic and eukaryotic homeostasis and cell signaling (Yoshikawa and Naito, 2000; Devasagayam et al., 2004; Gough and Cotter, 2011). However, when enriched in infected phagocytes by the activity of phagosomal myeloperoxidases and NO-oxidases, they represent effective antimicrobial measures. Accordingly, intracellular persisting mycobacteria have developed host defense escape mechanisms and the regulation of these is often linked to the expression of virulence factors.

Like other pathogenic mycobacteria, *Mycobacterium avium* ssp. *paratuberculosis* (MAP), the causative agent of Johne’s disease, a chronic, non-curable granulomatous inflammation of the ruminant intestine (Harris and Barletta, 2001; Lombard, 2011), is able to persist and multiply intracellularly in the phagosome of macrophages (Kuehnel et al., 2001). Specifically, MAP adapts to the intestinal environment by adjusting its metabolism to the host environment and responding to antimicrobial defense mechanisms indicated by enhanced expression of KatG and SodA (Weigoldt et al., 2011, 2013). Hence, MAP is clearly able to withstand the host innate defense response, but its enabling mechanisms are still unclear.

In general, oxidative stress response in bacteria is regulated by the major transcription factors OxyR and PerR (Zheng et al., 1999; Herbig and Helmann, 2001). OxyR belongs to the family of LysR-regulators and is not only found in Gram negative bacteria but also in some Gram positive bacteria (Oh et al., 2007). OxyR is a transcriptional activator under oxidizing conditions acting through direct interaction with the RNA polymerase α subunit (Kullik et al., 1995; Tao et al., 1995). PerR is a member of the ferric uptake regulator (FUR) family of metallo-regulators and functions as a peroxide-responsive repressor (Herbig and Helmann, 2001). PerR is found mostly in Gram positive bacteria where it seems to substitute for OxyR (Mongkolsuk and Helmann, 2002). Nevertheless, in some bacterial species PerR is present together with OxyR (van Vliet et al., 1999; Hahn et al., 2002; Tseng et al., 2003). To prevent damage by ROS, OxyR, and/or PerR regulate, amongst others, the expression of a catalase-peroxidase (*kat*) and an alkyl hydroperoxide reductase (*ahp*) in many bacteria (Christman et al., 1989; Storz and Altuvia, 1994; Bsat et al., 1996, 1998).

Iron plays a particular role in bacterial metabolism. On the one hand, iron is an essential structural and catalytic cofactor for many metabolic enzymes. On the other hand, iron excess in the cell is harmful due to the spontaneous generation of ROS by the Fenton reaction. Therefore, the bacterial intracellular iron homeostasis must be tightly regulated and often is closely linked to the response to oxidative stresses (Cornelis et al., 2011). In *Mycobacterium tuberculosis* (Mtb), three genes encoding putative iron-dependent regulatory proteins have been described (Cole et al., 1998): IdeR (Schmitt et al., 1995) and SirR (Hill et al., 1998), both belonging to the diptheria-toxin repressor (DtxR) family, as well as ferric uptake regulator A (FurA), a member of the FUR family (Lee and Helmann, 2007; Fillat, 2014).

IdeR is the global iron-dependent regulator in mycobacteria. As an iron-loaded homodimer, it controls the expression of about 50 genes in Mtb which are mostly involved in iron homeostasis, and in addition a number of virulence associated factors (Rodriguez et al., 2002). The *ide*R gene is essential in pathogenic mycobacteria like Mtb (Rodriguez et al., 2002) or MAP (Janagama et al., 2009), but not in saprophytic species such as *M. smegmatis* (Dussurget et al., 1996). Using a conditional *ide*R mutant, Pandey and Rodriguez (2014) recently demonstrated that IdeR is a factor important for the survival of Mtb in macrophages as well as for the infection of mice. SirR was originally identified in *Staphylococcus epidermidis* with a sequence identity to Mtb IdeR of 33% (Hill et al., 1998). The biological role of SirR homologs in pathogenic mycobacteria is still unclear.

The FUR family of transcriptional metallo-regulator proteins is widespread in bacteria and archaea (Fillat, 2014). The best characterized member of the FUR family, the *Escherichia coli* Fur protein, employs Fe^2+^ as co-repressor and acts as a global regulator, influencing the expression of more than 100 genes involved in iron homeostasis, intermediary metabolism, and oxidative stress response (McHugh et al., 2003). The classical mechanism of Fur regulation includes Fur-binding of Fe^2+^, formation of a metal-containing dimer which subsequently binds to a specific Fur-box found in the promoter sequences of the corresponding target genes (Bagg and Neilands, 1987). Recent studies indicated that the regulatory mechanisms governed by Fur exceed this classical repressor model. Thus, Fur homologs in different bacterial species can also act as metal-dependent positive regulators or even repress gene expression in the absence of the iron cofactor [apo-Fur repression; see review by Carpenter et al. (2009)]. In addition, a metal-independent activation of genes by apo-Fur was recently described (Butcher et al., 2012).

In all mycobacterial species sequenced so far *fur*A is located upstream of *kat*G. Furthermore, for some mycobacteria species, co-expression of *fur*A and *kat*G indicated an involvement of FurA in mycobacterial oxidative stress response. Indeed, it was proposed that, in mycobacteria, FurA could functionally replace the peroxide repressor PerR (Lucarelli et al., 2008). Nevertheless, the precise regulatory role of FurA and the regulation of stress response in mycobacteria are still not completely clear. One explanation for this might be the absence of a functional OxyR regulator in some mycobacterial species like those of the *M. tuberculosis complex* and *M. smegmatis*. In these species, it was found that FurA is involved in the oxidative stress response by regulating *kat*G (Pym et al., 2001; Zahrt et al., 2001) but there was no contribution of FurA to *ahp*C expression. In contrast, many other mycobacterial species, including the pathogens *M. avium* and *M. marinum,* harbor a functional *oxy*R gene (Deretic et al., 1995; Sherman et al., 1995). In these species it was shown that *ahp*C but not *kat*G is under control of OxyR (Dhandayuthapani et al., 1997; Pagan-Ramos et al., 1998). However, the regulation of *kat*G in these species has not been addressed. Overall, these data suggest a more complex regulatory network of oxidative stress response regulation in mycobacteria.

In the present work we aimed to analyze the relevance of mycobacterial FurA in iron homeostasis and stress response. We characterized the FurA regulon in MAP by construction and analysis of an isogenic MAPΔ*fur*A mutant. By this, we were able to clarify the influence of FurA^MAP^ in iron metabolism, stress response, and intracellular survival.

## MATERIALS AND METHODS

### CHEMICALS, BACTERIAL STRAINS, AND GROWTH CONDITIONS

All chemicals were purchased from Sigma–Aldrich (Munich, Germany) if not stated otherwise. Bacterial strains, oligonucleotides, plasmids, and phages are listed in Tables S1 and S2. *E. coli* strains were cultivated at 37°C in Luria-Bertani (LB) broth or on LB-agar supplemented with 100 μg/ml ampicillin or 100 μg/ml hygromycin, as required.

*Mycobacterium smegmatis* mc^2^ 155 and transformants were grown in LB media or on Middlebrook phage (MBP) agar (Supplementary Methods) supplemented with 100 μg/ml hygromycin if necessary. Liquid cultures were incubated at 37°C in a shaking incubator at 100 rpm. MAP DSM44135 wildtype (wt), MAPΔ*fur*A, and the complemented strain MAPΔ*fur*A^C^ were grown in Difco^TM^ Middlebrook 7H9 broth or on solid Middlebrook 7H10 agar (Beckton Dickinson, Franklin Lakes, NJ, USA) supplemented with mycobactin J (2 mg/l, Allied Monitor, Fayette, MO, USA), 2.5% glycerol and 10% OADC (0.06% oleic acid, 5% albumin, 2% dextrose, 0.085% NaCl, 0.003% catalase; MB-complete broth) and 50 μg/ml hygromycin and/or 50 μg/ml kanamycin, as required. Agar plates were sealed in plastic bags after inoculation and incubated at 37°C for 8–10 weeks. Liquid cultures were grown in Duran® laboratory glass bottles on a magnetic stirrer at 100 rpm and 37°C until they reached an OD_600_ of 1.0 and used for further experiments.

For growth kinetics, a starter culture was grown to an OD_600_ of 1.0 in MB-complete broth. Bacteria were harvested by centrifugation at 3,800 ×*g* for 15 min at 4°C, resuspended in MB-complete broth, singularized with glass beads (Ø 3 mm) by vortexing and inoculated into fresh medium to obtain an initial OD_600_ of 0.2. The OD_600_ of cultures was measured twice a week and bacteria were grown until they entered the stationary phase.

The response of MAPwt and MAPΔ*fur*A to oxidative stress was examined upon exposure of bacteria to hydrogen peroxide (H_2_O_2_). Cells were grown in MB-complete broth and harvested at an OD_600_ of 1.0 as described above. The bacterial pellet was washed twice with PBS and singularized cells were transferred to 50 ml Middlebrook 7H9 broth supplemented with 2 mg/l mycobactin, 2.5% glycerol, and 10% ADC lacking catalase (5% albumin, 2% dextrose, 0.085% NaCl; MB-cat broth) to obtain an OD_600_ of 1.0. H_2_O_2_ was added to a final concentration of 10 mM and cultures were exposed for 2 h at 37°C with gentle shaking (100 rpm) in Erlenmeyer flasks. Following, bacteria were harvested and immediately used for RNA-extraction.

### CONSTRUCTION OF A MAPΔ*fur*A STRAIN AND COMPLEMENTATION OF THE MUTANT STRAIN

Construction of a *fur*A (*map*1669c) deletion mutant of MAPwt was performed by specialized transduction according to Park et al. (2008) with minor modifications. A detailed protocol is given in the Supplementary Methods file.

For complementation of the mutant strain the *fur*A gene, including 88 bp of the upstream UTR, was amplified by PCR using primers oMAP-furA-K1/oMAP-furA-K2. The PCR product was digested with *Xba*I and *Hind*III and ligated into the corresponding sites of the mycobacterial integrative vector pMV306 (Stover et al., 1991), carrying a kanamycin resistance. The resulting plasmid pMAP-furA1101 was transformed into electro-competent MAPΔ*fur*A cells and plated on MB-complete agar with 50 μg/ml hygromycin and kanamycin. Successful deletion and integration of *fur*A was confirmed by PCR.

### CELL CULTURE OF MACROPHAGES AND SURVIVAL ASSAY OF INTRACELLULAR MYCOBACTERIA

The intracellular survival of MAPwt, MAPΔ*fur*A, and MAPΔ*fur*A^C^ was determined in the mouse macrophage cell line J774A.1 (Ralph et al., 1975). Macrophage cells were seeded in tissue culture dishes and were maintained in Dulbecco’s modified Eagle medium (DMEM) supplemented with 10% fetal calf serum (FCS), 1% glutamine, 100 units/ml penicillin, 100 μg/ml streptomycin at 37°C and 8% CO_2_. Twenty-four hours prior to infection, medium was changed to antibiotic-free DMEM. For infection experiments, MAP cells were grown to an OD_600_ of 1.0, harvested, singularized and stored in MB-complete broth containing 10% glycerol at -80°C until further usage. For infection, MAP cells were thawed on ice, diluted in antibiotic-free DMEM to an OD_600_ of 0.15 and incubated with the macrophages (2.0 × 10^6^ cells per cell culture dish) as described earlier (Kuehnel et al., 2001), resulting in a MOI of 10. The mean values of the initial inocula (five experiments) of all strains (MAPwt, MAPΔ*fur*A, MAPΔ*fur*A^C^) are shown in the Supplementary data files. At indicated time points, infected macrophages were washed twice with PBS and scraped off the plates in 1 ml PBS containing 0.1% SDS. J774A.1 cells were disrupted by passage through a 24-gauge needle. The homogenates as well as the infection inocula were 10-fold serial diluted in PBS and 25 μl of the 10^-4^ and 10^-5^ dilutions were plated on supplemented MB-complete agar plates in duplicate. Colony forming units (CFU) were determined after incubation for up to 10 weeks at 37°C and normalized to the initial inoculum.

### DETERMINATION OF REACTIVE OXYGEN SPECIES IN MACROPHAGE INFECTION EXPERIMENTS

Induction of ROS production by macrophages upon infection with MAPwt, MAPΔ*fur*A, and MAPΔ*fur*A^C^ was determined by use of CellRox® Deep Red (Life Technologies), according to the manufacturer’s protocol. In brief, macrophages were cultured and infected as described above and incubated for 2 h at 37°C/8% CO_2_ or treated with the chemical menadione as a positive control at a final concentration of 100 μM (Malorni et al., 1993). CellRox® Deep Red was added to a final concentration of 2.5 μM 30 min before harvesting and detection. Signals were measured at 660 nm by flow cytometry (Guava® easyCyte 8HT, Millipore, Billerica, MA, USA) and shown as mean fluorescence intensity (MFI) of four independent experiments.

### IMMUNOFLUORESCENCE AND CONFOCAL MICROSCOPY

Macrophages were seeded on coverslips and infected as described above. After 30 min and 2 h of infection, cells were fixed with 3% formaldehyde in PBS for 10 min, washed twice with PBS and treated with blocking buffer (PBS/1% BSA/10% FCS) for 20 min at room temperature. For staining of extracellular mycobacteria, coverslips were incubated with a 1:100 dilution of a polyclonal rabbit anti-MAP-HBHA serum (Supplementary Methods) in blocking buffer at room temperature in a humid chamber for 45 min. After washing with PBS, coverslips were incubated with goat anti-rabbit IgG coupled to Alexa Fluor® 488 (Life Technologies, Darmstadt, Germany) for 30 min and subsequently washed with PBS. To label intracellular mycobacteria, cells were permeabilized with Triton X-100 (0.1%) for 5 min at room temperature, washed in PBS, followed by incubation with a 1:100 dilution of polyclonal rabbit anti-MAP-HBHA serum in blocking buffer for 45 min at room temperature. After washing, coverslips were treated with goat anti-rabbit IgG coupled to Alexa Fluor® 568 (Life Technologies, Darmstadt, Germany) for 30 min. Coverslips were washed three times in PBS and, after one brief washing step with ddH_2_O, samples were mounted using ProLong® Gold with DAPI (Life Technologies, Darmstadt, Germany). Mounted samples were examined using a TCS SP5 confocal laser scanning microscope equipped with a 63×/1.4–0.6 NA HCX PL APO objective (Leica, Wetzlar, Germany). Image stacks were acquired using 1 Airy unit pinhole diameter in sequential imaging mode to avoid bleed through. Image stacks were deconvolved using Huygens® Essential (Scientific Volume Imaging, Hilversum, The Netherlands), maximum intensity projections were calculated for display purposes and adjusted for brightness and contrast using ImageJ/Fiji (Schindelin et al., 2012). After this labeling procedure, extracellular mycobacteria appear yellow, whereas intracellular mycobacteria are stained red.

### ANIMAL EXPERIMENT

Mouse infection experiments were approved by the Lower Saxony Federal State Office for Consumer Protection and Food Safety, Germany (reference number 08/1504). In each group, 9 female C57BL/6 mice aged 8 weeks (Charles River, Erkrath, Germany) were challenged intraperitoneally with an infection dose of 1 × 10^8^ bacteria of MAPwt and MAPΔ*fur*A in 200 μl Dulbecco’s Phosphate-Buffered Saline (DPBS, Life Technologies GmbH, Darmstadt, Germany). DPBS was used as negative control. Mice were sacrificed after 4 weeks, and liver and spleen were weighed. Bacteria were quantified by plating of homogenized tissue on MB-complete agar plates. Histology was performed in the Mouse Pathology Platform at HZI Braunschweig as recently described (Meißner et al., 2014; Suwandi et al., 2014).

### EXTRACTION OF NUCLEIC ACIDS AND QUANTITATIVE REAL-TIME PCR (qRT-PCR)

Genomic DNA was prepared as previously described (Stratmann et al., 2004). Plasmids were prepared using NucleoBond® AX kit (Macherey Nagel GmbH, Düren, Germany) according to the manufacturer’s protocol. Total RNA from bacteria was isolated according to Rustad et al. (2009) with minor modifications. In brief, bacterial pellets were resuspended in TRIzol® reagent, mechanically disrupted in a FastPrep® instrument (ThermoSavant, Carlsbad, CA, USA) and RNA was separated by chloroform, chloroform-isoamylalcohol (49:1 v/v) extraction, and precipitation with 2-propanol. Total RNA was treated twice with 50 U DNase I (Roche, Mannheim, Germany) according to the manufacturer’s protocol. Integrity and quality of RNA was determined by gel electrophoresis and spectrophotometric analysis using an Epoch instrument (Biotek, Bad Friedrichshall, Germany) at 260 nm.

For cDNA synthesis, 4 μg of DNA-depleted RNA were diluted with RNase free water up to a volume of 20 μl and incubated for 10 min at 70°C with 0.4 μg random primers (Promega, Madison, WI, USA). After 5 min cooling on ice, samples were split in two and mixed with 5x RT buffer, 10 mM dNTP’s, and either 200 U MMLV-superscript transcriptase (Promega) or buffer without transcriptase as a negative control. The mix was incubated for 1 h at 42°C, following incubation for 5 min at 85°C. Samples were diluted with 90 μl ddH_2_O and stored at -20°C for further analysis. 2.5 μl of each cDNA sample was mixed with 0.4 μM primer and 10 μl SYBR-Green Mix (Qiagen, Düsseldorf, Germany) in a total volume of 20 μl. Quantitative real-time PCR (qRT-PCR) experiments were performed using a Mx3005P qPCR system (Agilent Technologies, Santa Clara, CA, USA) with a thermal cycling profile as follows: segment 1, 20 min at 95°C, 1 cycle; segment 2, 45 s at 95°C, 1 min at 58°C, 1 min at 72°C, 45 cycles; segment 3, 1 min 95°C, 30 s 55°C, 30 s 95°C, 1 cycle. Each sample was analyzed in duplicate. Ct values were shown either as absolute data or normalized to the housekeeping gene *gap* and expressed as fold change to the untreated control (ΔΔct). All oligonucleotide primer pairs (Table S1) were tested for efficacy with a serial dilution of genomic DNA.

### RNA DEEP SEQUENCING AND ANALYSIS

RNA deep sequencing was used to determine the regulon of FurA in MAP. Sequencing libraries of MAPwt and MAPΔ*fur*A RNA, of three independent samples each, were prepared and sequenced using 36 bp single-ends sequencing on a Genome Analyzer IIx (Illumina, San Diego, CA, USA) or 50 bp single-ends sequencing on a HiSeq2500 instrument (Illumina), respectively. In brief, libraries of 300 bp were prepared according the manufacturer’s instructions “Preparing Samples for Sequencing of mRNA” (Illumina) and “Protocol for ScriptSeq^TM^ v2 RNA-Seq Library Preparation” (Epicentre Biotechnologies, Madison, WI, USA). Quality of the libraries was validated using an Agilent Bioanalyzer (Agilent Technologies) following the manufacturer’s instruction. Cluster generation was performed using the Illumina cluster station; sequencing on the Genome Analyzer IIx or HiSeq2500 followed a standard protocol. The fluorescent images were processed to sequences and transformed to FastQ format using the Genome Analyzer Pipeline Analysis software 1.8.2 (Illumina). The sequence output was controlled for general quality features using the fastq-mcf tool of ea-utils (Aronesty, 2013) and was mapped against the genome sequence of the reference strain MAP-K10 (AE016958) using BWA version 0.7.5 (Li and Durbin, 2009) and SAMtools (Li et al., 2009) for storing nucleotide sequence alignments. Strain MAP-K10 was used as a reference strain as the genome of MAP DSM44135 has not been sequenced yet. Raw data sets are available in the European Nucleotide Archive Repository ^1^. For data analyses, reads of the 50 bp sequencing were clipped to 36 bp reads. Following, all sequences were computed with Rockhopper tool (McClure et al., 2013). Genes with a *q*-value ≤0.01 were considered as significantly differentially expressed and genes with raw counts in all replicates were included for further analysis (Table S3). The fold change was calculated as the expression value of MAPwt/MAPΔ*fur*A.

### BIOINFORMATICS AND STATISTICS

Differentially expressed genes identified by Rockhopper analysis were further processed with Blast2Go tool (Conesa et al., 2005) and NCBI blastx ^2^ as well as tuberculosis database ^3^ to investigate functions of putative proteins. Additionally, the putative proteins were clustered into orthologous groups (COG), based on the genome of MAP-K10 ^4^. Data are expressed as mean ± SEM, and statistical analyses were performed using GraphPad Prism 5.03 (GraphPad, San Diego, CA, USA). Depending on the experiment, either the non-parametric *t*-test (Mann–Whitney) or one-way ANOVA test (Kruskal–Wallis) were used. A *p*-value of <0.05 between samples and controls was considered as statistically significant.

Prediction of putative FurA binding sites in MAP (NCBI accession No. NC_002944) was performed by FIMO analysis (Bailey et al., 2009). Initially, we generated a consensus sequence for FurA binding by comparison of sequences located -70/+30 bp up-/downstream of predicted FurA translation start sites in Mtb (NC_000962, Rv1909c), *M. bovis* BCG pasteur (NC_008769, BCG_1948c), *M. avium* ssp. *avium* 104 (NC_008595, MAV_2752), MAP (NC_002944, MAP_1669c), and *M. smegmatis* (NC_008596, MSMEG_6383) with MEME Suite (Bailey et al., 2009). The resulting binding motif T[CT]TTGACT[CG][AG]TTCCA[GA]A[AT]AA[GT][GT][GC][ACG][GC][TG]CAT[AT] was subsequently submitted to FIMO analysis. Nucleotides in brackets are variable, single nucleotides are conserved.

## RESULTS

### CHARACTERIZATION OF A MAPΔ*fur*A DELETION MUTANT

Ferric uptake regulator A (FurA) has been described as a global regulator in* E. coli* and was proposed to be involved in oxidative stress response and regulation of iron homeostasis (Zheng et al., 1999). In mycobacteria, iron homeostasis is maintained by IdeR, and FurA is assumed to take part in general stress response due to its genomic co-localization and its (partly) co-transcription with *kat*G. However, the regulatory role of FurA has not yet been fully understood.

**FIGURE 1:**
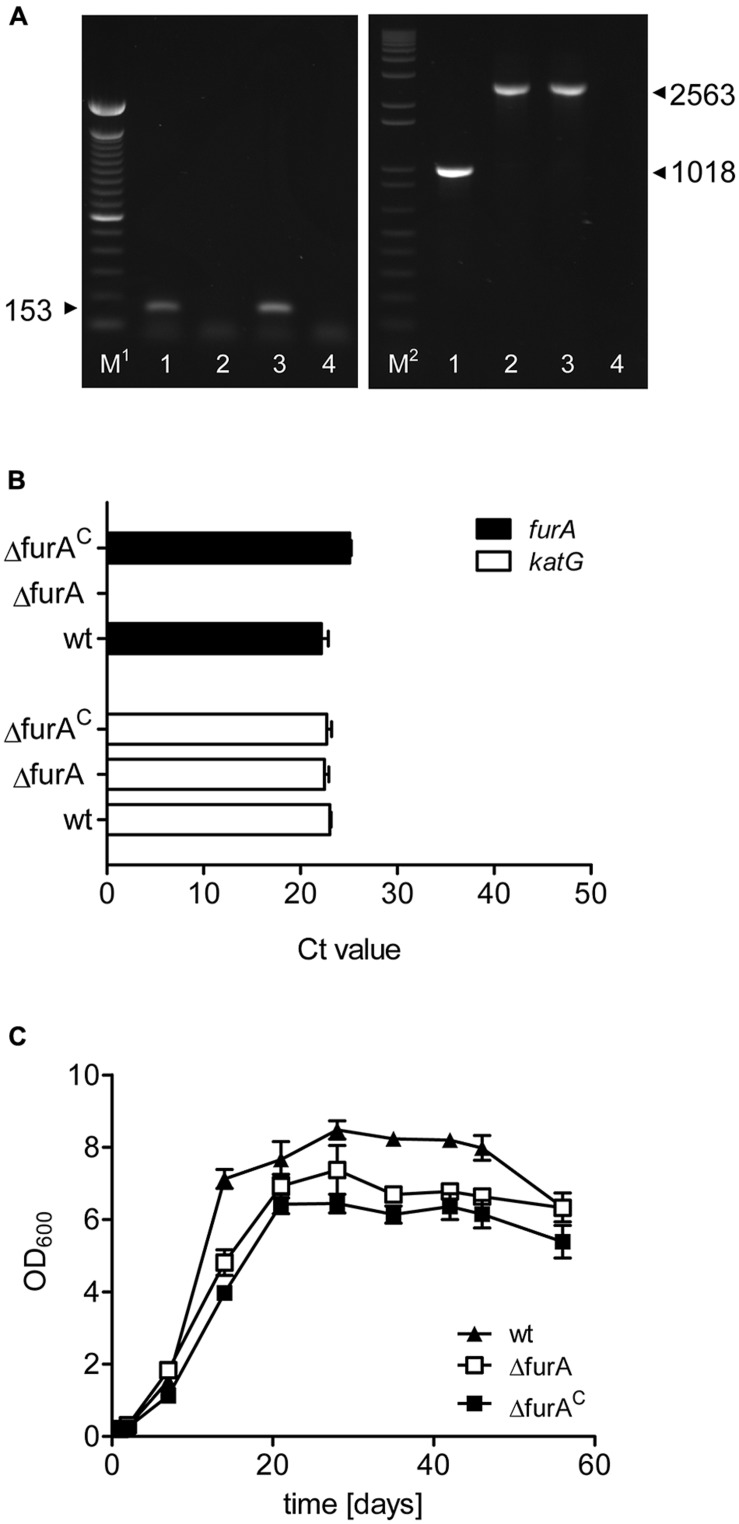
**Characterization of a MAPΔ*fur*A mutant. (A)** Confirmation of deletion and complementation of *fur*A. Chromosomal DNA was prepared from MAPwt (lanes 1), MAPΔ*fur*A (lanes 2), and MAPΔ*fur*A^C^ (lanes 3) grown in MB-complete broth to an OD_600_ of 1.0 and was subsequently tested in PCR for the presence of *fur*A (left panel) with primers oRTfurA1 fw/rev and hygromycin (right panel) with primers oTKfurAKatGfw/oRTlppsrev. A negative control with water only is shown in lanes 4. M^1^ = 100 bp DNA ladder (Invitrogen), M^2^ = 1 kb plus DNA ladder. Samples were run on a 1.5% agarose gel. **(B)** Total RNA was extracted from all strains grown to an OD_600_ of 1.0 in MB-complete broth. Expression of *fur*A (black bars) and the adjacent gene *kat*G (white bars) was analyzed by qRT-PCR. Results are expressed as mean Ct values of three independent experiments. The level of the housekeeping gene *gap* for all samples was 19.27 ± 0.4060. **(C)** Growth of MAPwt (filled triangles), MAPΔ*fur*A (open rectangle), and the complemented strain MAPΔ*fur*A^C^ (filled rectangle) was monitored for 60 days by measurement of the optical density (OD_600_) in MB-complete broth. Each growth experiment was conducted in triplicate for each strain. Shown are representative results of one out of two independent experiments.

To gain insight into the role of FurA in MAP, we generated a *fur*A (*map*1669c) deletion mutant MAPΔ*fur*A by specialized transduction. In addition, a complemented strain was constructed by introducing the integrative plasmid pFurA-MAP1101, harboring the *fur*A gene under control of its own promoter, into the Δ*fur*A mutant strain, resulting in strain MAPΔ*fur*A^C^. Deletion of the *fur*A gene in MAPΔ*fur*A and complementation of MAPΔ*fur*A^C^ were confirmed by PCR (**Figure 1A**). Expression of *fur*A and the adjacent gene *kat*G were analyzed by qRT-PCR. *fur*A transcripts were detected in MAP wildtype (MAPwt) and MAPΔ*fur*A^C^ but not in MAPΔ*fur*A. The expression levels of *kat*G were similar in all strains (**Figure 1B**), indicating that the genetic manipulation had no polar effect on the expression of downstream located genes. Next we compared growth of MAPwt, MAPΔ*fur*A, and MAPΔ*fur*A^C^ in MB-complete broth. As shown in **Figure 1C**, all strains exhibited similar growth kinetics. Growth of MAPΔ*fur*A and MAPΔ*fur*A^C^ was slightly reduced in comparison to MAPwt, and both strains reached stationary growth phase at slightly lower OD_600_ than MAPwt. These effects might be due to the antibiotic supplements in the growth media.

### DETERMINATION OF THE FurA REGULON OF MAP

To analyze the regulatory role of FurA in MAP, we prepared RNA from MAPwt and MAPΔ*fur*A grown to an OD_600_ of 1.0 in MB-complete broth and performed RNA deep sequencing. Gene expression profiles of three independent replicates of each strain were analyzed with the Rockhopper analysis tool (McClure et al., 2013), and genes with a *q*-value of ≤0.01 were considered as significantly differentially expressed. Differences in gene expression were calculated as fold change of expression values calculated by Rockhopper analysis (MAPΔ*fur*A vs. MAPwt).

In total, we identified 48 genes, which were differentially expressed in the mutant strain. Compared to the wt strain, 13 genes were higher (**Table 1**) and 35 lower (**Table 2**) expressed in MAPΔ*fur*A. Clustering of putative proteins encoded by the differentially expressed genes into orthologous groups (COG) revealed that almost 40% of the differentially expressed genes were assigned to metabolism, 20.75% to cellular processes and signaling, 9.43% belong to the group of poorly characterized proteins, and 30.19% were not assigned to any COG (**Tables 1** and **2**). No genes related to iron homeostasis, such as siderophores (mycobactin) and siderophore uptake systems (Neilands, 1992; Escolar et al., 1998; McHugh et al., 2003) or iron storage proteins *bfr*, *fnt*A (Abdul-Tehrani et al., 1999; Wildermann et al., 2004), were affected by the *fur*A deletion in MAP.

**Table 1:** Genes higher expressed in MAPΔ*fur*A compared to MAPwt.

		Orthologous genes (% similarity)					
RCN^a^	Annotation	Mtb	MAA	*q*-Value^b^	Fold change^c^	Putative function^d^	COG^e^
–	MAP0771	–	MAV_0960 (93.8)	<0.0001	8.23	Membrane protein	–
–	MAP0772	–	MAV_0961 (100)	<0.0001	7.00	Membrane protein	–
–	MAP0773	Rv2193 (45.88)	MAV_0962 (93.22)	<0.0001	11.63	Cytochrome c oxidase subunit iii	C
–	MAP1587c	Rv2471 (45.07)	MAV_2842 (91.81)	<0.0001	21.34	Alpha-amylase	G
ahpD	MAP1588c	Rv2429 (84.57)	MAV_2840 (99.43)	<0.0001	21.70	Alkyl hydroperoxide reductase	S
ahpC	MAP1589c*	Rv2428 (93.33)	MAV_2839 (100)	<0.0001	29.87	Alkyl hydroperoxide reductase c protein ahpc	O
–	MAP1741c	Rv2026c (68.58)	MAV_2507 (94.21)	0.00145	4.11	Universal stress protein	T
–	MAP1742c	Rv2026c (84.49)	MAV_2506 (74.4)	<0.0001	8.33	Universal stress protein	T
–	MAP1743c	Rv2032 (64.64)	MAV_2505 (90.09)	<0.0001	24.33	NAD(P)H nitroreductase	–
cysH_1	MAP2036	Rv2392 (83.85)	MAV_2153 (98.78)	0.00058	6.43	Phosphoadenosine phosphosulfate reductase	EH
–	MAP2037	Rv2393 (73.24)	MAV_2152 (91.19)	<0.0001	6.74	Cobalamin (vitamin B12) biosynthesis CbiX protein	S
fdxC_1	MAP2039	Rv1177 (89.62)	MAV_2150 (99.07)	<0.0001	7.72	Ferredoxin	C
–	MAP3677	Rv2242 (51.28)	MAV_4933 (81.93)	0.00069	4.75	PucR family transcriptional regulator	TQ

*^a^Reference common name*.

*^b^*q*-value of differentially expressed genes MAPΔ*fur*A vs. wildtype (wt) calculated by Rockhopper analysis. A *q*-value <0.01 is considered as significant*.

*^c^gene expression values of MAPΔ*fur*A divided by gene expression values of the wt from RNA-sequencing*.

*^d^Putative function based on Blast2Go, NCBI Blast analysis or TB database search*.

*^e^Functional classification of proteins has been performed by use of COG database with MAP-K10 as a reference (http://www.ncbi.nlm.nih.gov/sutils/coxik.cgi?gi=380). (C) Energy production and conversion, (E) Amino acid transport and metabolism, (G) Carbohydrate transport and metabolism, (H) Coenzyme transport and metabolism, (O) Posttranslational modification, protein turnover, chaperones, (Q) Secondary metabolites biosynthesis, transport and catabolism, (S) Function unknown, (T) Signal transduction mechanisms*.

*predicted FurA binding sites present in the promoter region, identified by MEME Suite.

**Table 2:** Genes lower expressed in MAPΔ*fur*A compared to MAPwt.

		Orthologous genes (% similarity)			
RCN^a^	Annotation	Mtb	MAA	*q*-Value^b^	Fold change^c^	Putative function^d^	COG^e^
–	MAP0047c	Rv0040c (90.9)	MAV_0054 (73.79)	<0.0001	-12.91	Proline-rich 28 kDa antigen	–
–	MAP0079	Rv2002 (50.81)	MAV_0087 (95.12)	<0.0001	-8.00	Oxidoreductase	IQR
–	MAP0081*	Rv0063 (76.76)	MAV_0089 (85.61)	<0.0001	-7.67	Oxidoreductase	C
–	MAP0130	–	MAV_0124 (91.48)	<0.0001	-8.37	Hypothetical protein MAP0130	T
–	MAP0337	Rv1648 (52.83)	MAV_0364 (89.63)	<0.0001	-6.00	Membrane protein	–
–	MAP0847*	Rv1754c (83.96)	MAV_1035 (100)	<0.0001	-19.77	Conserved protein	–
PE	MAP1003c	Rv1040c (75.55)	MAV_1179 (54.03)	<0.0001	-12.40	PPE family protein	N
–	MAP1203	Rv1477 (76.48)	MAV_3301 (91.13)	<0.0001	-11.63	Invasion protein	M
–	MAP1204	Rv1478 (72.76)	MAV_3300 (86.88)	<0.0001	-9.58	Invasin 1	M
–	MAP1272c	Rv1566c (83.58)	MAV_3208 (76.04)	0.00059	-6.19	nlpc p60 family protein	M
–	MAP1388	–	MAV_3090 (74.19)	<0.0001	-4.67	Hypothetical protein MAP1388	–
–	MAP1418c	Rv3821 (56.25)	MAV_3059 (84.67)	0.00950	-4.00	Hypothetical protein MAP1418c	–
–	MAP1570	Rv1861 (50.49)	MAV_2858 (65.16)	<0.0001	-5.52	Membrane protein	–
–	MAP1639c	Rv0854 (75.86)	MAV_2785 (99.32)	0.00074	-4.21	Cyclase/dehydratase	I
furA	MAP1669c*	Rv1909c (88.02)	MAV_2752 (100)	<0.0001	-52.80	FurA	P
–	MAP1706	Rv1987 (73.1)	MAV_2710 (100)	<0.0001	-10.38	Chitinase	G
–	MAP1967c	Rv2223c (78.81)	MAV_2243 (88.43)	0.00014	-5.86	Exported protease	–
–	MAP2114c	Rv1968 (61.83)	MAV_2063 (90.27)	<0.0001	-6.67	Virulence factor mce family protein	Q
–	MAP2117c	Rv3501c (66.0)	MAV_0946 (66.53)	0.00103	-4.69	ABC transporter permease	Q
–	MAP2168c	Rv2376c (55.82)	MAV_2017 (90.35)	<0.0001	-11.59	Low molecular weight antigen mtb12	–
fdxC_2	MAP2607c	Rv1177 (96.22)	MAV_1316 (100)	<0.0001	-11.90	Ferredoxin	C
–	MAP2642	Rv1660 (64.75)	MAV_1280 (96.27)	<0.0001	-11.50	Polyketide synthase	Q
–	MAP3123c	–	MAV_3943 (74.1)	<0.0001	-5.20	Hypothetical protein	–
–	MAP3310	Rv3209 (80.68)	MAV_4157 (100)	0.00471	-4.73	Membrane protein	–
–	MAP3421c*	–	MAV_4276 (99)	0.00114	-4.00	Diguanylate cyclase phosphodiesterase	–
–	MAP3796	Rv0309 (78.99)	MAV_4853 (96.95)	<0.0001	-9.24	Conserved exported protein	S
–	MAP3800	Rv0312 (68.43)	MAV_2177 (52.59)	<0.0001	-10.89	Hypothetical protein	O
–	MAP3812c	Rv0116c (80.39)	MAV_4834 (100)	0.00030	-4.60	Hypothetical protein MAP3812c	S
–	MAP3868	Rv0754 (64.7)	MAV_4778 (91.69)	0.00011	-4.40	PE-PGRS family protein	G
–	MAP4204	Rv1888c (68.75)	MAV_4428 (88.82)	0.00382	-3.78	Transmembrane protein	–
–	MAP4206c	–	MAV_4427 (75.39)	<0.0001	-24.00	Eﬄux ABC transporter, permease protein	M
–	MAP4208	Rv3003c (40.8)	MAV_4424 (88.85)	<0.0001	-6.71	Acetolactate synthase	EH
–	MAP4209	–	MAV_4423 (100)	<0.0001	-6.73	Hypothetical protein MAP4209	–
–	MAP4210	–	MAV_4422 (99.37)	<0.0001	-10.00	3-oxoacyl-acp synthase	I
–	MAP4211	Rv3772 (46.15)	MAV_4421 (89.77)	<0.0001	-8.75	Histidinol-phosphate aminotransferase	E

*^a^Reference common name*.

*^b^*q*-value of differentially expressed genes MAPΔ*fur*A vs. wt calculated by Rockhopper analysis. A *q*-value <0.01 is considered as significant*.

*^c^gene expression values of MAPΔ*fur*A divided by gene expression values of the wt from RNA-sequencing*.

*^d^Putative function based on Blast2Go, NCBI Blast analysis or TB database search*.

*^e^Functional classification of proteins has been performed by use of COG database with MAP-K10 as a reference (http://www.ncbi.nlm.nih.gov/sutils/coxik.cgi?gi=380). (C) Energy production and conversion, (E) Amino acid transport and metabolism, (G) Carbohydrate transport and metabolism, (H) Coenzyme transport and metabolism, (I) Lipid transport and metabolism, (M) Cell wall/membrane/envelope biogenesis, (N) Cell motility, (O) Posttranslational modification, protein turnover, chaperones, (P) Inorganic ion transport and metabolism, (Q) Secondary metabolites biosynthesis, transport and catabolism, (R) General function prediction only, (S) Function unknown, (T) Signal transduction mechanisms*.

*predicted FurA binding sites present in the promoter region, identified by MEME Suite.

Among the 13 higher expressed genes, 11 were organized in 4 putative operons. The majority of these genes encodes for proteins predicted to be involved in stress response or redox processes. Within this group, the gene cluster *map*1589c–1587c, encoding for two alkyl hydroperoxide reductases involved in oxidative stress and drug resistance as well as intracellular survival in Mtb (Sherman et al., 1999; Master et al., 2002), and a NAD(P)H nitroreductase (*map*1743c), which has been shown to be associated with detoxification of nitroaromatic components in Mtb (Purkayastha et al., 2002), exhibited the most significant expression changes (**Table 1**).

The group of lower expressed genes is comprised of 35 genes. Aside from *fur*A, this group consists of two putative transporters, five genes predicted to be involved in metabolism, three stress response-associated genes and 13 genes were of unknown function. The remaining 11 genes can be associated to virulence which is represented by two putative invasion proteins (*map*1203/1204), two putative antigens (*map*0047c/*map*2168c), one Pro-Pro-Glu protein (PPE; *map*1003c), one PE-PGRS glycin-rich protein (*map*3868), one nlpc p60 protein (*map*1272c), and one mammalian cell entry (mce) family protein (*map*2114c) as well as three other enzymes (*map*1706, *map*1967c, *map*3421c) for cell entry and persistence. Altogether, *map*0047c, *map*0847 (conserved protein), and *map*4206c (putative eﬄux ABC transporter) showed the most significant differences in expression compared to the wt (**Table 2**). In addition, the 5′ UTRs of *map*1589c (*ahp*C), *map*0081 (putative oxidoreductase), *map*0847, and *map*3421c (putative diguanylate cyclase phosphodiesterase) featured a predicted FurA binding site (**Tables 1** and **2**). Overall, these analyses indicated a considerable involvement of MAP FurA in the adaptation of MAP to the host cell environment and general stress response.

**FIGURE 2:**
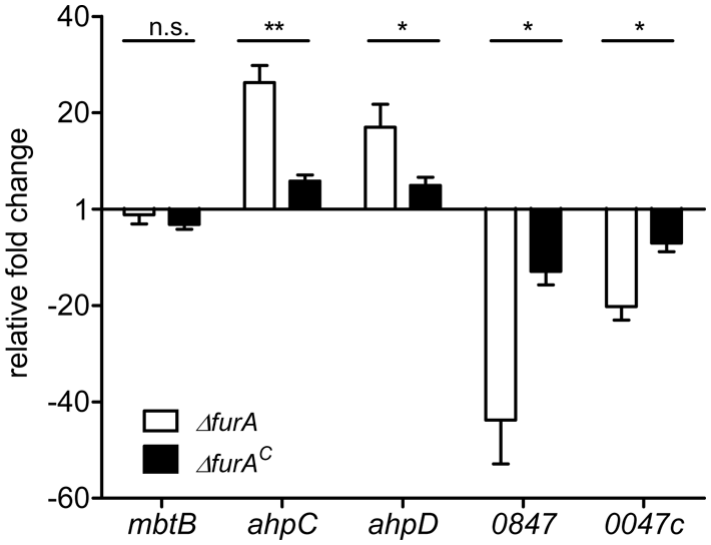
**Functional complementation of FurA-regulated genes.** MAPwt, MAPΔ*fur*A and the complemented strain MAPΔ*fur*A^c^ were grown in MB-complete broth to an OD_600_ of 1.0. Total RNA was extracted, followed by cDNA synthesis and qRT-PCR analysis. Expression of genes *mbt*B, *ahp*C (*map*1589c), *ahp*D (*map*1588c), *map*0847, and *map*0047c in MAPΔ*fur*A (white bars) and MAPΔ*fur*A^c^ (black bars) was compared to transcription levels in MAPwt. Three independent experiments were analyzed, qRT-PCR was performed in duplicate for each sample, normalized to the housekeeping gene *gap* and expressed as fold change compared to the MAPwt (mean ± SEM). Statistical analysis was performed using Mann–Whitney test with *p < 0.05, **p < 0.005.

To confirm the results of our RNA deep sequencing analyses and to further specify the involvement of FurA in regulation, we analyzed the transcription of *ahp*C and *ahp*D (*map*1589c/1588c, higher expressed in MAPΔ*fur*A), and of *map*0847 and *map*0047c (lower expressed in MAPΔ*fur*A) by qRT-PCR. MAPwt, MAPΔ*fur*A, and the complemented strain MAPΔ*fur*A^C^ were grown in MB-complete broth to an OD_600_ of 1.0, RNA was extracted and analyzed by qRT-PCR. As a control, we included the *mbt*B gene (*map*2177c) which has been shown to be regulated iron-dependently by IdeR in Mtb (Gold et al., 2001) and MAP (Janagama et al., 2009). As expected, the expression of *mbt*B was similar in all strains. In agreement with our RNA sequencing analyses, *ahp*C, *ahp*D, *map*0847, and *map*0047c were up- and down-regulated in the mutant, respectively. In the complemented mutant MAPΔ*fur*A^C^ expression levels were mostly restored to wt levels (**Figure 2**). The differences to the wt strain might be due to the slightly lower expression level of *fur*A in MAPΔ*fur*A^C^ compared to MAPwt (**Figure 1B**). Overall, these results confirm the regulatory influence of FurA on expression of *ahp*C, *ahp*D, *map*0847, and *map*0047c.

### *ahp* EXPRESSION IN MAP IS HYDROGEN PEROXIDE DEPENDENT

AhpC and AhpD are metal dependent alkyl hydroperoxide reductases and constitute an antioxidant system important in reduction and neutralization of a wide range of organic hydroperoxides and peroxynitrite (Wood et al., 2003). Genes encoding for AhpC/D have been shown to be induced by peroxide stress mediated by OxyR in other mycobacteria (Dhandayuthapani et al., 1997). However, in MAP these genes seem to be regulated by FurA which resembles PerR mediated activation in *Bacillus* and *Campylobacter* (van Vliet et al., 1999; Helmann et al., 2003). Thus, we exposed MAPwt and MAPΔ*fur*A cultures without catalase supplement to oxidative stress by addition of 10 mM H_2_O_2_ for 2 h and analyzed *ahp*D expression by qRT-PCR. As a negative control we used the FurB dependent but FurA independent regulated *mpt*A (*map*3736c) gene (Eckelt et al., 2014).

**FIGURE 3:**
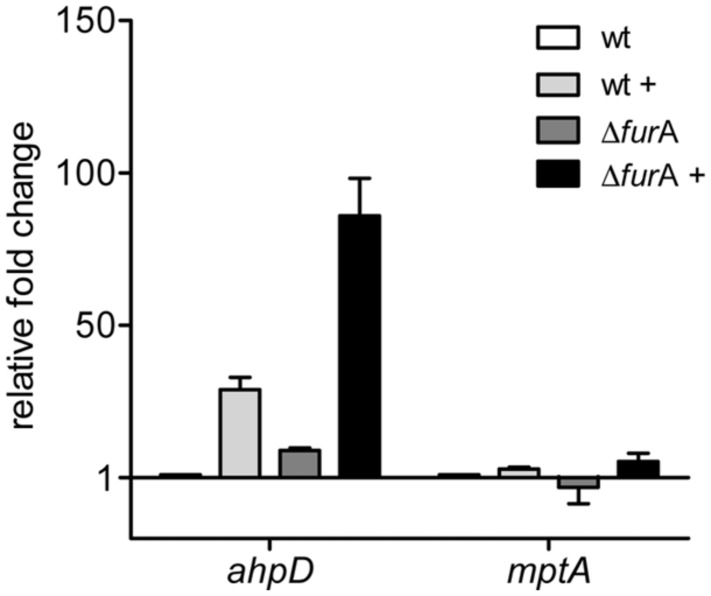
**Response of the FurA regulated alkyl hydroperoxide reductase system AhpCD to oxidative stress.** MAPwt and MAPΔ*fur*A cultures were grown in MB-complete broth, harvested at OD_600_ of 1.0, washed, transferred to MB-cat broth and incubated for 2 h with or without 10 mM H_2_O_2_. RNA was isolated and used for cDNA synthesis. Gene expression analysis by qRT-PCR was performed for the genes *ahp*D (*map*1588c) and *mpt*A (*map*3736c) in untreated (MAPwt, white bars; Δ*fur*A, dark gray bars) and H_2_O_2_ treated samples (MAPwt, light gray bars; Δ*fur*A, black bars). Results were normalized to the housekeeping gene *gap* and are expressed as fold change compared to the untreated MAPwt control (mean ± SEM, *n* = 3).

As shown in **Figure 3**, expression of *ahp*D in MAPΔ*fur*A was increased compared to the wt and supports RNA-sequencing data. Upon peroxide treatment, both strains responded with an induction of *ahp*D expression. However, the relative fold change in MAPΔ*fur*A was considerably higher than in MAPwt compared to the untreated control.

In contrast *mpt*A expression was similar in all strains at both conditions (standard/oxidative stress).

These data on the one hand side indicate that repression of *ahp*C/D by FurA can be relieved by peroxide. On the other hand they demonstrate that an additional peroxide sensing regulatory activity, most probably by OxyR or a OxyR-like regulator, co-regulates *ahp*C/D expression in MAP.

### MAPΔ*fur*A EXHIBITS HIGHER RESISTANCE TO INTRACELLULAR KILLING IN MACROPHAGES BUT FurA IS DISPENSABLE DURING CHRONIC INFECTION

**FIGURE 4:**
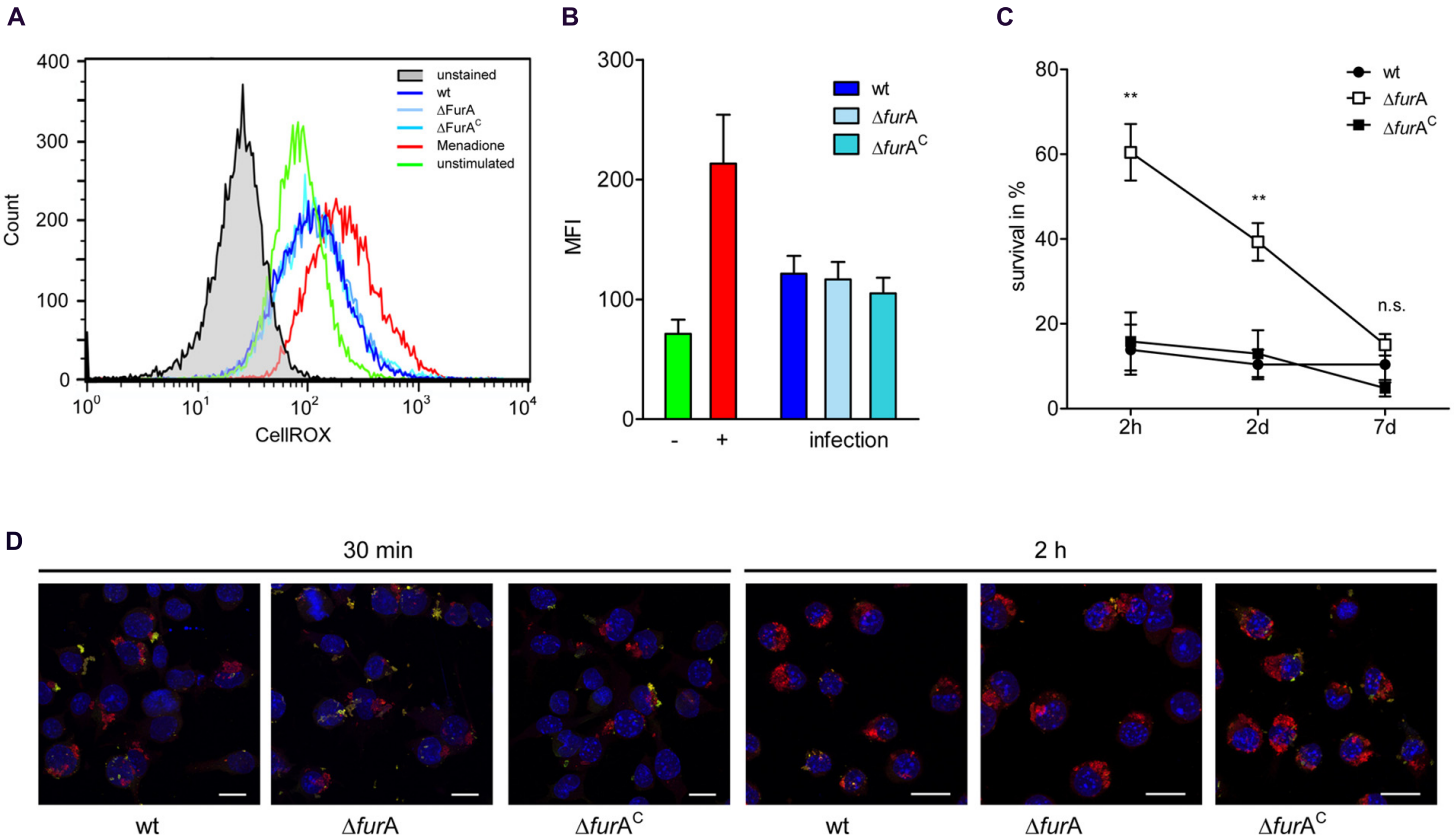
**Induction of oxidative burst in macrophages and survival of MAP strains in macrophages.** J774A.1 macrophages (Mø) were grown in DMEM and infected with an MOI of 10 with MAPwt, MAPΔ*fur*A and the complemented strain MAPΔ*fur*A^C^ grown in MB-complete broth to an OD_600_ of 1.0. Mø were infected as described above for 1.5 h or treated with 100 μM menadione (+). CellROX Deep Red was then added to a final concentration of 2.5 mM for 30 min. **(A)** Histograms showing the production of ROS measured by CellROX Deep Red staining of unstimulated cells (green histogram) or infected Mø with MAPwt (dark blue histogram), MAPΔ*fur*A (light blue histogram), and the complemented strain MAPΔ*fur*A^C^ (turquoise histogram). Mø treated with 100 μM menadione were used as a positive control (red histogram). Unstained cells are shown in the gray histogram. One representative experiment out of four is shown. **(B)** Fluorescence of untreated (-, green bar), menadione treated (+, red bar), and with MAPwt (dark blue bar), MAPΔ*fur*A (light blue bar) or MAPΔ*fur*A^C^ (turquoise bar) infected macrophages was determined by FACS analysis. Shown are the results of four independent experiments (mean fluorescence intensity, MFI ± SEM). **(C)** Mø were infected as described above and incubated for 2 h, 2 and 7 days. Cells were disrupted and the lysate was diluted prior plating. 25 μl of the dilutions were plated in duplicate on MB-complete agar and incubated for up to 10 weeks. Five independent experiments were conducted, CFU of lysates and of the respective inocula used for macrophage infection were determined, and mycobacterial survival was expressed as percent to the inoculum. Statistical analysis was performed using one-way ANOVA (Kruskal–Wallis) with **p < 0.005. **(D)** Confocal maximum intensity projections of a double immunofluorescence staining of J774A.1 macrophages infected for 30 min or 2 h with MAPwt, MAPΔ*fur*A, and MAPΔ*fur*A^C^. Extracellular mycobacteria were labeled with anti-MAP-HBHA and green fluorescent Alexa Fluor^®^ 488, intracellular bacteria with anti-MAP-HBHA and orange fluorescent Alexa Fluor^®^ 568. Nuclei were stained blue with DAPI. Extracellular bacteria appear yellow and intracellular appear red. Bar represents 15 μm.

It has been shown that *ahp*C/D are necessary for defense against ROS and RNS in the mycobacterial phagosome (Sherman et al., 1995; Wilson et al., 1998; Master et al., 2002). By this pathogenic mycobacteria are able to survive within macrophages, enabling metabolic adaptation to the phagosomal compartment which is necessary for persistence. The above data showed that FurA is involved in the regulation of MAP response to oxidative stress, as indicated by the regulation of enzymes involved in neutralization of oxygen and nitrogen radicals [*ahp*C/D, NAD(P)H nitroreductase]. Therefore, we aimed to address the role of FurA for intracellular survival of MAPwt, MAPΔ*fur*A, and MAPΔ*fur*A^C^ in macrophages. First we confirmed that the MAP strains are exposed to ROS after being taken up by the macrophages. For this, J774A.1 macrophages were infected with MAPwt, MAPΔ*fur*A, and MAPΔ*fur*A^C^. After 1.5 h of infection, cells were treated with the reagent CellROX Deep Red for 30 min. This dye fluoresces upon oxidation and allows the detection of ROS. As positive control we included macrophages treated (+) with menadione (**Figure 4B**) which is known to induce the generation of ROS in eukaryotic cells (Malorni et al., 1993). As shown in **Figures 4A,B**, the MFI measured by FACS analysis in macrophages infected with MAPwt, MAPΔ*fur*A and the complemented mutant strain was higher than in untreated (-) macrophages (**Figure 4B**), indicating the generation of ROS induced by all MAP strains. Next, cells were infected with MAPwt, MAPΔ*fur*A, and MAPΔ*fur*A^C^ as described above and bacterial survival was determined after 2 h, 2 and 7 days by CFU-counting after serial dilution plating. After 2 h and 2 days infection we found significantly higher numbers of viable MAPΔ*fur*A cells compared to MAPwt (**Figure 4C**). Complementation of MAPΔ*fur*A almost completely restored the phenotype to wt level. Confocal analyses of macrophages infected with the different MAP strains for 30 min or 2 h after inside-outside staining of bacteria showed no differences in mycobacterial uptake by and adhesion to the macrophages (**Figure 4D**). These findings clearly exclude a better phagocytosis of MAPΔ*fur*A which could account for the initial higher survival of MAPΔ*fur*A in the macrophages. Interestingly, the percentage of viable MAPΔ*fur*A cells decreased over time from ∼60% (of the initial inoculum) at 2 h to ∼40% at day 2 and to ∼18% at day 7. In contrast, no change in survival was visible in the wt and complement strain, indicating that the mutant was also being killed (**Figure 4C**). Calculating the survival rates at day 2 and 7 relative to the number of bacteria taken up by the macrophages after the 2 h infection period revealed that MAPΔ*fur*A even though being more resistant to initial killing, is much more efficiently killed during the time course of macrophage infection (data not shown). Overall, these data indicate that MAPΔ*fur*A can better resist the initial killing by the macrophage most probably caused by oxidative stress but is susceptible to macrophage defense mechanisms present at later time points of infection or less adapted to the phagosomal life style.

**FIGURE 5:**
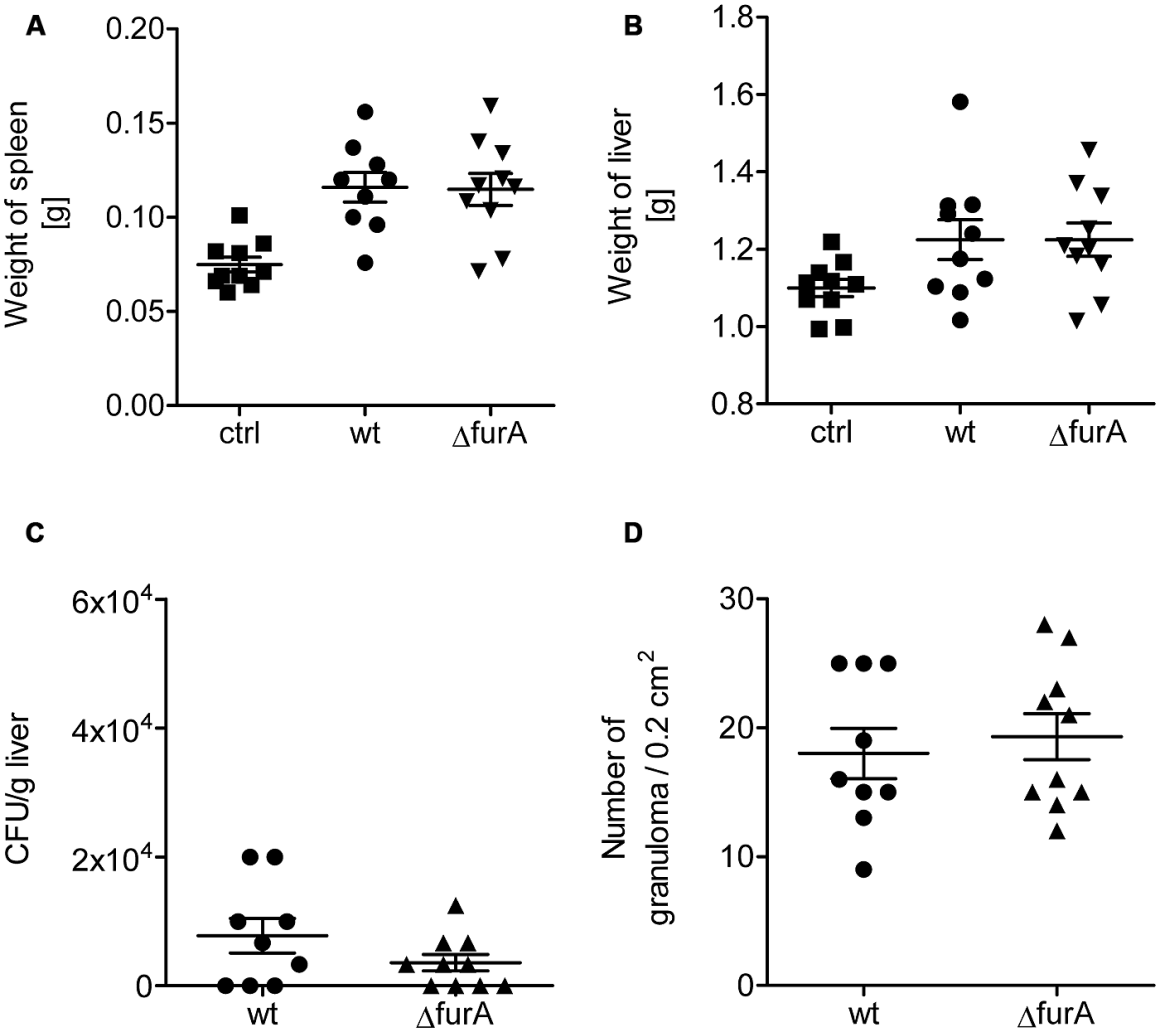
**Biological fitness of MAPwt and MAPΔ*fur*A in infected mice.** Eight weeks old female C57BL/6 mice were infected with MAPwt (circles) and MAPΔ*fur*A (triangle), grown to an OD_600_ of 1.0, with an infection dose of 1 × 10^8^ cells and sacrificed 4 weeks later. The control group was treated with DPBS buffer (squares). Weight of spleen **(A)**, liver **(B)**, CFU detection of bacteria from liver **(C),** and the amount of granuloma in the liver **(D)** were chosen as read-out parameters. The results represent the mean ± SEM of one animal experiment with 9 mice in each group.

Hence, we analyzed the role of FurA for survival in the host. For this, C57BL/6 mice were infected intraperitoneally with 1 × 10^8^ bacteria of exponentially grown cultures (OD_600_ of 1.0) and were sacrificed 4 weeks post infection. As shown in **Figure 5**, no significant differences in weight of liver and spleen, as well as number of bacteria or granuloma in the liver upon infection with MAPwt and MAPΔ*fur*A were detected. However, as a trend we observed lower CFU numbers in the livers of MAPΔ*fur*A infected cells. Overall, the lack of FurA in MAP did not alter survival rates compared to the wt and suggests that FurA might be dispensable for MAP-infection in the mouse model.

## DISCUSSION

Bacterial iron homeostasis is tightly regulated and closely linked to the cellular response against cytotoxic effects due to the generation of ROS by free iron ions via the Fenton reaction (Cornelis et al., 2011). Accordingly, FurA was first identified in *E. coli* as global transcriptional regulator influencing expression of genes associated with iron acquisition and storage, but was later also shown to be involved in the regulation of genes activated by cellular stress (Dubrac and Touati, 2000). In mycobacteria, *fur*A is located immediately upstream of *kat*G encoding a catalase-peroxidase. Both genes are co-induced by peroxide treatment (Master et al., 2001; Milano et al., 2001; Jang et al., 2009). From this and the higher resistance of a *fur*A mutant of the fast growing *M. smegmatis* to peroxide stress (Zahrt et al., 2001), it was proposed that in mycobacterial species, FurA is involved in the oxidative stress response regulation (Lucarelli et al., 2008). However, the precise role of FurA in pathogenic mycobacteria has not yet been elucidated.

In the present study, we characterized the role of *fur*A in MAP. By RNAseq analyses, we were able to dissect a specific involvement of FurA in the regulation of the MAP response to reactive nitrogen and oxygen radicals, in intracellular survival, and in the expression of predicted virulence-associated genes. Most interestingly, unlike in many other bacterial species, where Fur regulates the expression of iron uptake and storage systems (Bagg and Neilands, 1987; Bsat et al., 1998; Abdul-Tehrani et al., 1999), FurA did not participate in the regulation of iron homeostasis in MAP. The involvement of FurA in modulating stress response and mechanisms for evading host cell defense as well as for invasion and infection is emphasized as 30 of the 48 regulated genes have been shown by works of other groups to be differentially expressed in MAP after infection of macrophages or *in vivo* infection of cattle (9 M/O, 21 *in vivo*; Wu et al., 2007; Cossu et al., 2012; Ghosh et al., 2013) and 26 of these genes have been found to be associated to stress response, as they were significantly regulated when MAP was exposed to acidic pH, nitrosamine, hydrogen peroxide, or heat (Wu et al., 2007; Kawaji et al., 2010; Cossu et al., 2012).

Our transcriptomic data also revealed that the FurA regulon includes genes with repressed and with activated transcription. Expression of a defined group of 13 genes encoding proteins of general stress response and enzymes necessary for the generation of reductive activity to detoxify oxygen radicals was higher in MAPΔ*fur*A and is therefore repressed by FurA. For instance *map*1589c (*ahp*C) and *map*1588c (*ahp*D) were found to be induced in MAP upon oxidative stress (Kawaji et al., 2010) and during macrophage infection (Ghosh et al., 2013). Additionally, we found a peroxide dependent regulation of these genes by FurA in MAP. The alkyl hydroperoxide reductases AhpC and AhpD of the peroxireductase family (Prx) form an antioxidant system that is necessary for the reduction and neutralization of a wide range of organic hydroperoxides and peroxynitrite (Wood et al., 2003) to protect structures particularly sensitive to peroxide-mediated damage, such as lipids and nucleic acids (Halliwell and Gutteridge, 1984). A regulation of *ahp* genes by the Fur-like regulator PerR has been described for other bacteria, e.g. *Bacillus subtilis* and *Campylobacter jejuni* (van Vliet et al., 1999; Helmann et al., 2003). The regulatory mechanism bases on the property of mononuclear iron proteins such as FurA to lose repressive activity during both O_2_^-^ and H_2_O_2_ stress by oxidation of Fe^2+^ to Fe^3+^. In addition to the missing activation of iron acquisition systems it supports our hypothesis of the role of FurA as ROS sensing regulator in MAP. CysH has been shown to contribute to protection against oxidative and nitrosative stress in Mtb (Senaratne et al., 2006) and many other FurA-repressed genes encode for enzymes involved in reactions liberating reducing activity in form of H^+^ ions.

Beyond this, FurA actively contributes to the expression of a subset of 35 genes many of which are suggested to be associated to virulence or intracellular survival. A major group of FurA-activated genes comprises genes encoding invasins (MAP1204/1204), antigens (MAP2168c, Webb et al., 1998), proteins of the Pro-Pro-Glu (PPE) family (MAP1003c, Akhter et al., 2012), PE-PGRS glycine-rich proteins, (MAP3868, Iantomasi et al., 2012), nlpc p60 (MAP1272c, Parthasarathy et al., 2012), and a mce family protein (MAP2114c, Gioffre et al., 2005) as well as several enzymes for cell entry, and it has been demonstrated for other bacteria, that homologs to these genes are important virulence factors (Kulasakara et al., 2006; Frederiksen et al., 2013). Interestingly, also some proteins involved in electron transfer were affected which suggest that FurA contributes to the maintenance of the canonical endogenous redox balance. Finally we found, that *kat*G expression was not affected by *fur*A deletion. Despite the fact, that a co-transcription of both genes and regulation of *kat*G by FurA was suggested for other mycobacteria species (Master et al., 2001; Pym et al., 2001; Zahrt et al., 2001), we could not confirm this for MAP. Hence, *kat*G might be regulated independently of FurA as proposed for *Mycobacterium* sp. strain JC1 DSM 3803 (Lee et al., 2009) or other bacteria (Italiani et al., 2011).

The particular role of FurA for virulence in mycobacteria has not been shown yet. Like other pathogenic mycobacteria, MAP is able to infect and survive in macrophages. Therefore, MAP must be able to react to the antimicrobial macrophage response such as reactive oxygen radicals. Indeed, our macrophage infection experiments with MAPwt, MAPΔ*fur*A, and MAPΔ*fur*A^C^ showed the induction of ROS formation in the host cell. When monitoring the intracellular fate of MAPwt, MAPΔ*fur*A, and MAPΔ*fur*A^C^, we observed a significantly higher percentage of viable MAPΔ*fur*A cells compared to the wt and MAPΔ*fur*A^C^ during the initial phase of infection. The higher percentage resulted from higher survival most probably given by an enhanced resistance of MAPΔ*fur*A to oxidative stress mediated by the expression of FurA-repressed genes. Enhanced resistance to oxidative stress was also described for *C. jejuni* and *M. smegmatis*: in these species, deletion of *per*R and *fur*A, respectively, resulted in a hyper resistancy to peroxide stress (van Vliet et al., 1999; Pym et al., 2001). In contrast, the survival rate of MAPΔ*fur*A was considerably lower compared to the wt and MAPΔ*fur*A^C^, indicated by killing of the mutant over time. This suggests that genes activated by FurA are needed for the adaptation of MAP to the macrophage phagosomal microenvironment.

In the intestinal tissue of the infected host, metabolic adaptation, and oxidative stress defense dominate the metabolism of MAP (Weigoldt et al., 2011, 2013). In contrast to the macrophage infections, the *fur*A deletion seems not to affect MAP survival after 4 weeks intraperitoneal MAP infection of mice. Histological staining did not show significant differences in the number of granuloma and granuloma size as well as composition (data not shown). This suggests that the phenotype of MAPΔ*fur*A is not advantageous when MAP has to counteract a complex immune response or has to metabolically adapt to the host environment. Nevertheless, we cannot exclude that FurA might play a role in a later stage of infection or effects of the FurA deletion might only be notable very early after infection.

## CONCLUSION

In conclusion, in the presented study, we determined for the first time the regulon of FurA in a pathogenic mycobacterial species. Our data provide novel insights into the function of FurA in mycobacteria. They suggest two different physiological roles executed by FurA. Firstly, FurA acts as a repressor for a selective group of genes involved in the response to oxidative stresses. During oxidative stress it might operate as an iron-based sensor of ROS and, therefore, might be functionally closely related to PerR. Secondly, FurA contributes to activation of a second group of genes, many of which are suggested to contribute to MAP long term survival in macrophages. To conclusively investigate the role of FurA in virulence, however, infection experiments in animals developing clinical disease are required.

## AUTHOR CONTRIBUTIONS

Ralph Goethe, Gerald-F. Gerlach, and Jochen Meens designed the experiments; Thorsten Meißner constructed the mutant, Thorsten Meißner and Kristin Laarmann accomplished mutant complementation, Michael Jarek performed RNA sequencing; Thorsten Meißner, Elke Eckelt, and Kristin Laarmann characterized the mutant and the complemented strain; Elke Eckelt performed stress experiments, Andreas Nerlich and Kristin Laarmann performed the macrophage experiments, Ralph Goethe and Siegfried Weiss designed the mouse infection experiments, Thorsten Meißner performed the mouse infections. Thorsten Meißner, Elke Eckelt, Jochen Meens, Ralph Goethe analyzed data; and Elke Eckelt, Ralph Goethe, Gerald-F. Gerlach, and Jochen Meens wrote the paper.

## Conflict of Interest Statement

The authors declare that the research was conducted in the absence of any commercial or financial relationships that could be construed as a potential conflict of interest.

## Footnotes

1 http://www.ebi.ac.uk/ena/data/view/PRJEB7916

2 http://blast.ncbi.nlm.nih.gov/Blast.cgi

3 http://tbdb.org

4 http://www.ncbi.nlm.nih.gov/sutils/coxik.cgi?gi=380

## References

[B1] Abdul-Tehrani H. Hudson A. J. Chang Y.-S. Timms A. R. Hawkins C. Williams J. M. (1999). Ferritin mutants of *Escherichia coli* are iron deficient and growth impaired, and fur mutants are iron deficient. *J. Bacteriol.* 181 1415–1428. 10049371 10.1128/jb.181.5.1415-1428.1999 PMC93529

[B2] Akhter Y. Ehebauer M. T. Mukhopadhyay S. Hasnain S. E. (2012). The PE/PPE multigene family codes for virulence factors and is a possible source of mycobacterial antigenic variation: perhaps more? *Biochemie* 94 110–116 10.1016/j.biochi.2011.09.026 22005451

[B3] Aronesty E. (2013). Comparison of sequencing utility programs. *Open Bioinformatics J.* 7 1–8 10.2174/1875036201307010001

[B4] Bagg A. Neilands J. B. (1987). Ferric uptake regulation protein acts as a repressor, employing iron (II) as a cofactor to bind the operator of an iron transport operon in *Escherichia coli. Biochemistry* 26 5471–5477 10.1021/bi00391a039 2823881

[B5] Bailey T. L. Boden M. Buske F. A. Frith M. Grant C. E. Clementi L. (2009). MEME SUITE: tools for motif discovery and searching. *Nucleic Acids Res.* 37 W202–W208 10.1093/nar/gkp335 19458158 PMC2703892

[B6] Bsat N. Chen L. Helmann J. D. (1996). Mutation of the *Bacillus subtilis* alkyl hydroperoxide reductase (ahpCF) operon reveals compensatory interactions among hydrogen peroxide stress genes. *J. Bacteriol.* 178 6579–6586. 8932315 10.1128/jb.178.22.6579-6586.1996 PMC178545

[B7] Bsat N. Herbig A. Casillas-Martinez L. Setlow P. Helmann J. D. (1998). *Bacillus subtilis* contains multiple Fur homologues: identification of the iron uptake (Fur) and peroxide regulon (PerR) repressors. *Mol. Microbiol.* 29 189–198 10.1046/j.1365-2958.1998.00921.x 9701813

[B8] Butcher J. Sarvan S. Brunzelle J. S. Couture J. F. Stintzi A. (2012). Structure and regulon of *Campylobacter jejuni* ferric uptake regulator Fur define apo-Fur regulation. *Proc. Natl. Acad. Sci. U.S.A.* 109 10047–10052 10.1073/pnas.1118321109 22665794 PMC3382491

[B9] Carpenter B. M. Whitmire J. M. Merrell D. S. (2009). This is not your mother’s repressor: the complex role of fur in pathogenesis. *Infect. Immun.* 77 2590–2601 10.1128/IAI.00116-09 19364842 PMC2708581

[B10] Christman M. F. Storz G. Ames B. N. (1989). OxyR, a positive regulator of hydrogen peroxide-inducible genes in *Escherichia coli* and *Salmonella typhimurium*, is homologous to a family of bacterial regulatory proteins. *Proc. Natl. Acad. Sci. U.S.A.* 86 3484–3488 10.1073/pnas.86.10.3484 2471187 PMC287162

[B11] Cole S. T. Brosch R. Parkhill J. Garnier T. Churcher C. Harris D. (1998). Deciphering the biology of *Mycobacterium tuberculosis* from the complete genome sequence. *Nature* 393 537–544 10.1038/31159 9634230

[B12] Conesa A. Gotz S. Garcia-Gomez J. M. Terol J. Talon M. Robles M. (2005). Blast2GO: a universal tool for annotation, visualization and analysis in functional genomics research. *Bioinformatics* 21 3674–3676 10.1093/bioinformatics/bti610 16081474

[B13] Cornelis P. Wei Q. Andrews S. C. Vinckx T. (2011). Iron homeostasis and management of oxidative stress response in bacteria. *Metallomics* 3 540–549 10.1039/c1mt00022e 21566833

[B14] Cossu A. Sechi L. A. Zanetti S. Rosu V. (2012). Gene expression profiling of *Mycobacterium avium* subsp. *paratuberculosis* in simulated multi-stress conditions and within THP-1 cells reveals a new kind of interactive intramacrophage behaviour. *BMC Microbiol.* 12:87 10.1186/1471-2180-12-87 PMC3416667 22646160

[B15] Deretic V. Philipp W. Dhandayuthapani S. Mudd M. H. Curcic R. Garbe T. (1995). *Mycobacterium tuberculosis* is a natural mutant with an inactivated oxidative-stress regulatory gene: implications for sensitivity to isoniazid. *Mol. Microbiol.* 17 889–900 10.1111/j.1365-2958.1995.mmi_17050889.x 8596438

[B16] Devasagayam T. P. Tilak J. C. Boloor K. K. Sane K. S. Ghaskadbi S. S. Lele R. D. (2004). Free radicals, and antioxidants in human health: current status, and future prospects. *J. Assoc. Physicians India* 52 794–804. 15909857

[B17] Dhandayuthapani S. Mudd M. Deretic V. (1997). Interactions of OxyR with the promoter region of the oxyR and ahpC genes from *Mycobacterium leprae* and *Mycobacterium tuberculosis* *J. Bacteriol.* 179 2401–2409. 10.1128/jb.179.7.2401-2409.1997 PMC178979 9079928

[B18] Dubrac S. Touati D. (2000). Fur positive regulation of iron superoxide dismutase in *Escherichia coli*: functional analysis of the sodB promoter. *J. Bacteriol.* 182 3802–3808 10.1128/JB.182.13.3802-3808.2000 10850997 PMC94553

[B19] Dussurget O. Rodriguez M. Smith I. (1996). An ideR mutant of *Mycobacterium smegmatis* has derepressed siderophore production and an altered oxidative-stress response. *Mol. Microbiol.* 22 535–544 10.1046/j.1365-2958.1996.1461511.x 8939436

[B20] Eckelt E. Jarek M. Frömke C. Meens J. Goethe R. (2014). Identification of a lineage specific zinc responsive genomic island in *Mycobacterium avium* ssp. *paratuberculosis. BMC Genomics* 15:1076 10.1186/1471-2164-15-1076 PMC4298942 25481572

[B21] Escolar L. Perez-Martin J. de Lorenzo V. (1998). Binding of the fur (ferric uptake regulator) repressor of *Escherichia coli* to arrays of the GATAAT sequence. *J. Mol. Biol.* 283 537–547 10.1006/jmbi.1998.2119 9784364

[B22] Fillat M. F. (2014). The FUR (ferric uptake regulator) superfamily: diversity and versatility of key transcriptional regulators. *Arch. Biochem. Biophys.* 546C, 41–52 10.1016/j.abb.2014.01.029 24513162

[B23] Frederiksen R. F. Paspaliari D. K. Larsen T. Storgaard B. G. Larsen M. H. Ingmer H. (2013). Bacterial chitinases and chitin-binding proteins as virulence factors. *Microbiology* 159 833–847 10.1099/mic.0.051839-0 23519157

[B24] Ghosh P. Wu C. W. Talaat A. M. (2013). Key role for the alternative sigma factor, sigH, in the intracellular life of *Mycobacterium avium* subsp. *paratuberculosis* during macrophage stress *Infect. Immun.* 81 2242–2257 10.1128/IAI.01273-12 23569115 PMC3676012

[B25] Gioffre A. Infante E. Aguilar D. Santangelo M. P. Klepp L. Amadio A. (2005). Mutation in mce operons attenuates *Mycobacterium tuberculosis* virulence. *Microbes Infect.* 7 325–334 10.1016/j.micinf.2004.11.007 15804490

[B26] Gold B. Rodriguez G. M. Marras S. A. Pentecost M. Smith I. (2001). The *Mycobacterium tuberculosis* IdeR is a dual functional regulator that controls transcription of genes involved in iron acquisition, iron storage and survival in macrophages. *Mol. Microbiol.* 42 851–865 10.1046/j.1365-2958.2001.02684.x 11722747

[B27] Gough D. R. Cotter T. G. (2011). Hydrogen peroxide: a Jekyll and Hyde signalling molecule. *Cell Death Dis.* 2:e213 10.1038/cddis.2011.96 PMC3219092 21975295

[B28] Hahn J. S. Oh S. Y. Roe J. H. (2002). Role of OxyR as a peroxide-sensing positive regulator in *Streptomyces coelicolor* A3(2). *J. Bacteriol.* 184 5214–5222 10.1128/JB.184.19.5214-5222.2002 12218006 PMC137946

[B29] Halliwell B. Gutteridge J. M. (1984). Oxygen toxicity, oxygen radicals, transition metals and disease. *Biochem. J.* 219 1–14. 6326753 10.1042/bj2190001 PMC1153442

[B30] Harris N. B. Barletta R. G. (2001). *Mycobacterium avium* subsp. *paratuberculosis* in Veterinary Medicine. *Clin. Microbiol. Rev.* 14 489–512 10.1128/CMR.14.3.489-512.2001 11432810 PMC88986

[B31] Helmann J. D. Wu M. F. Gaballa A. Kobel P. A. Morshedi M. M. Fawcett P. (2003). The global transcriptional response of *Bacillus subtilis* to peroxide stress is coordinated by three transcription factors. *J. Bacteriol.* 185 243–253 10.1128/JB.185.1.243-253.2003 12486061 PMC141929

[B32] Herbig A. F. Helmann J. D. (2001). Roles of metal ions and hydrogen peroxide in modulating the interaction of the *Bacillus subtilis* PerR peroxide regulon repressor with operator DNA. *Mol. Microbiol.* 41 849–859 10.1046/j.1365-2958.2001.02543.x 11532148

[B33] Hill P. J. Cockayne A. Landers P. Morrissey J. A. Sims C. M. Williams P. (1998). SirR, a novel iron-dependent repressor in *Staphylococcus epidermidis*. *Infect. Immun.* 66 4123–4129. 9712757 10.1128/iai.66.9.4123-4129.1998 PMC108495

[B34] Iantomasi R. Sali M. Cascioferro A. Palucci I. Zumbo A. Soldini S. (2012). PE_PGRS30 is required for the full virulence of *Mycobacterium tuberculosis*. *Cell Microbiol.* 14 356–367 10.1111/j.1462-5822.2011.01721.x 22050772

[B35] Italiani V. C. da Silva Neto J. F. Braz V. S. Marques M. V. (2011). Regulation of catalase-peroxidase KatG is OxyR dependent and Fur independent in *Caulobacter crescentus*. *J. Bacteriol.* 193 1734–1744 10.1128/JB.01339-10 21257767 PMC3067672

[B36] Janagama H. K. Senthilkumar T. M. Bannantine J. P. Rodriguez G. M. Smith I. Paustian M. L. (2009). Identification and functional characterization of the iron-dependent regulator (IdeR) of *Mycobacterium avium* subsp. *paratuberculosis. Microbiology* 155 3683–3690 10.1099/mic.0.031948-0 PMC2888127 19684064

[B37] Jang H. J. Nde C. Toghrol F. Bentley W. E. (2009). Microarray analysis of *Mycobacterium bovis* BCG revealed induction of iron acquisition related genes in response to hydrogen peroxide. *Environ. Sci. Technol.* 43 9465–9472 10.1021/es902255q 19924887

[B38] Kawaji S. Zhong L. Whittington R. J. (2010). Partial proteome of *Mycobacterium avium* subsp. *paratuberculosis* under oxidative and nitrosative stress. *Vet. Microbiol.* 145 252–264 10.1016/j.vetmic.2010.03.025 20413229

[B39] Kuehnel M. P. Goethe R. Habermann A. Mueller E. Rohde M. Griffiths G. (2001). Characterization of the intracellular survival of *Mycobacterium avium* ssp. *paratuberculosis:* phagosomal pH and fusogenicity in J774 macrophages compared with other mycobacteria. *Cell. Microbiol.* 3 551–566 10.1046/j.1462-5822.2001.00139.x 11488816

[B40] Kulasakara H. Lee V. Brencic A. Liberati N. Urbach J. Miyata S. (2006). Analysis of *Pseudomonas aeruginosa* diguanylate cyclases and phosphodiesterases reveals a role for bis-(3′-5′)-cyclic-GMP in virulence. *Proc. Natl. Acad. Sci. U.S.A.* 103 2839–2844 10.1073/pnas.0511090103 16477007 PMC1413825

[B41] Kullik I. Toledano M. B. Tartaglia L. A. Storz G. (1995). Mutational analysis of the redox-sensitive transcriptional regulator OxyR: regions important for oxidation and transcriptional activation. *J. Bacteriol.* 177 1275–1284. 7868602 10.1128/jb.177.5.1275-1284.1995 PMC176734

[B42] Lee H.-I. Yoon J.-H. Nam J.-S. Kim Y.-M. Ro Y.-T. (2009). Cloning, expression and characterization of the catalase-peroxidase (KatG) gene from a fast-growing *Mycobacterium* sp. strain JC1 DSM 3803. *J. Biochem.* 147 511–522 10.1093/jb/mvp197 19933836

[B43] Lee J. W. Helmann J. D. (2007). Functional specialization within the Fur family of metalloregulators. *Biometals* 20 485–499 10.1007/s10534-006-9070-7 17216355

[B44] Li H. Durbin R. (2009). Fast and accurate short read alignment with Burrows-Wheeler transform. *Bioinformatics.* 25 1754–1760 10.1093/bioinformatics/btp324 19451168 PMC2705234

[B45] Li H. Handsaker B. Wysoker A. Fennell T. Ruan J. Homer N. (2009). The Sequence Alignment/Map format and SAMtools. *Bioinformatics* 25 2078–2079 10.1093/bioinformatics/btp352 19505943 PMC2723002

[B46] Lombard J. E. (2011). Epidemiology and economics of paratuberculosis. *Vet. Clin. North Am. Food Anim. Pract.* 27 525–535 10.1016/j.cvfa.2011.07.012 22023831

[B47] Lucarelli D. Vasil M. L. Meyer-Klaucke W. Pohl E. (2008). The metal-dependent regulators FurA and FurB from *Mycobacterium tuberculosis*. *Int. J. Mol. Sci.* 9 1548–1560 10.3390/ijms9081548 19169435 PMC2630230

[B48] Malorni W. Iosi F. Santini M. T. Testa U. (1993). Menadione-induced oxidative stress leads to a rapid down-modulation of transferrin receptor recycling. *J. Cell Sci.* 106(Pt 1), 309–318. 8270633 10.1242/jcs.106.1.309

[B49] Master S. S. Springer B. Sander P. Boettger E. C. Deretic V. Timmins G. S. (2002). Oxidative stress response genes in *Mycobacterium tuberculosis*: role of ahpC in resistance to peroxynitrite and stage-specific survival in macrophages. *Microbiology* 148 3139–3144. 12368447 10.1099/00221287-148-10-3139

[B50] Master S. Zahrt T. C. Song J. Deretic V. (2001). Mapping of *Mycobacterium tuberculosis* katG promoters and their differential expression in infected macrophages. *J. Bacteriol.* 183 4033–4039 10.1128/JB.183.13.4033-4039.2001 11395468 PMC95287

[B51] McClure R. Balasubramanian D. Sun Y. Bobrovskyy M. Sumby P. Genco C. A. (2013). Computational analysis of bacterial RNA-Seq data. *Nucleic Acids Res.* 41:e140 10.1093/nar/gkt444 PMC3737546 23716638

[B52] McHugh J. P. Rodriguez-Quinones F. Abdul-Tehrani H. Svistunenko D. A. Poole R. K. Cooper C. E. (2003). Global iron-dependent gene regulation in *Escherichia coli*. A new mechanism for iron homeostasis. *J. Biol. Chem.* 278 29478–29486 10.1074/jbc.M303381200 12746439

[B53] Meißner T. Eckelt E. Basler T. Meens J. Heinzmann J. Suwandi A. (2014). The *Mycobacterium avium* ssp. *paratuberculosis* specific *mptD* gene is required for maintaining of the metabolic homeostasis necessary for full virulence in mouse infections. *Front. Cell Infect. Microbiol.* 4:110 10.3389/fcimb.2014.00110 PMC4132290 25177550

[B54] Milano A. Forti F. Sala C. Riccardi G. Ghisotti D. (2001). Transcriptional regulation of furA and katG upon oxidative stress in *Mycobacterium smegmatis*. *J. Bacteriol.* 183 6801–6806 10.1128/JB.183.23.6801-6806.2001 11698368 PMC95520

[B55] Mongkolsuk S. Helmann J. D. (2002). Regulation of inducible peroxide stress responses. *Mol. Microbiol.* 45 9–15 10.1046/j.1365-2958.2002.03015.x 12100544

[B56] Neilands J. B. (1992). Mechanism and regulation of synthesis of aerobactin in *Escherichia coli* K12 (pColV-K30). *Can. J. Microbiol.* 38 728–733 10.1139/m92-119 1393837

[B57] Oh S. Y. Shin J. H. Roe J. H. (2007). Dual role of OhrR as a repressor and an activator in response to organic hydroperoxides in *Streptomyces coelicolor*. *J. Bacteriol.* 189 6284–6292 10.1128/JB.00632-07 17586628 PMC1951921

[B58] Pagan-Ramos E. Song J. McFalone M. Mudd M. H. Deretic V. (1998). Oxidative stress response and characterization of the oxyR-ahpC and furA-katG loci in *Mycobacterium marinum. J. Bacteriol.* 180 4856–4864. 10.1128/jb.180.18.4856-4864.1998 PMC107510 9733688

[B59] Pandey R. Rodriguez G. M. (2014). IdeR is required for iron homeostasis and virulence in *Mycobacterium tuberculosis*. *Mol. Microbiol.* 91 98–109 10.1111/mmi.12441 24205844 PMC3902104

[B60] Park K. T. Dahl J. L. Bannantine J. P. Barletta R. G. Ahn J. Allen A. J. (2008). Demonstration of allelic exchange in the slow-growing bacterium *Mycobacterium avium* subsp. *paratuberculosis*, and generation of mutants with deletions at the pknG, relA, and lsr2 loci. *Appl. Environ. Microbiol.* 74 1687–1695 10.1128/AEM.01208-07 18192416 PMC2268294

[B61] Parthasarathy G. Lun S. Guo H. Ammerman N. C. Geiman D. E. Bishai W. R. (2012). Rv2190c, an NlpC/P60 family protein, is required for full virulence of *Mycobacterium tuberculosis*. *PLoS ONE* 7:e43429 10.1371/journal.pone.0043429 PMC3432046 22952680

[B62] Purkayastha A. McCue L. A. McDonough K. A. (2002). Identification of a *Mycobacterium tuberculosis* putative classical nitroreductase gene whose expression is coregulated with that of the acr gene within macrophages, in standing versus shaking cultures, and under low oxygen conditions. *Infect. Immun.* 70 1518–1529 10.1128/IAI.70.3.1518-1529.2002 11854240 PMC127740

[B63] Pym A. S. Domenech P. Honore N. Song J. Deretic V. Cole S. T. (2001). Regulation of catalase-peroxidase (KatG) expression, isoniazid sensitivity and virulence by furA of *Mycobacterium tuberculosis*. *Mol. Microbiol.* 40 879–889 10.1046/j.1365-2958.2001.02427.x 11401695

[B64] Ralph P. Prichard J. Cohn M. (1975). Reticulum cell sarcoma: an effector cell in antibody-dependent cell-mediated immunity. *J. Immunol.* 114 898–905. 1089721

[B65] Rodriguez G. M. Voskuil M. I. Gold B. Schoolnik G. K. Smith I. (2002). ideR, an essential gene in *Mycobacterium tuberculosis*: role of IdeR in iron-dependent gene expression, iron metabolism, and oxidative stress response. *Infect. Immun.* 70 3371–3381 10.1128/IAI.70.7.3371-3381.2002 12065475 PMC128082

[B66] Rustad T. R. Roberts D. M. Liao R. P. Sherman D. R. (2009). Isolation of mycobacterial RNA. *Methods Mol. Biol.* 465 13–21 10.1007/978-1-59745-207-6_2 20560069

[B67] Schindelin J. Arganda-Carreras I. Frise E. Kaynig V. Longair M. Pietzsch T. (2012). Fiji: an open-source platform for biological-image analysis. *Nat. Methods* 9 676–682 10.1038/nmeth.2019 22743772 PMC3855844

[B68] Schmitt M. P. Predich M. Doukhan L. Smith I. Holmes R. K. (1995). Characterization of an iron-dependent regulatory protein (IdeR) of *Mycobacterium tuberculosis* as a functional homolog of the diphtheria toxin repressor (DtxR) from *Corynebacterium diphtheriae*. *Infect. Immun.* 63 4284–4289. 7591059 10.1128/iai.63.11.4284-4289.1995 PMC173608

[B69] Senaratne R. H. De Silva A. D. Williams S. J. Mougous J. D. Reader J. R. Zhang T. (2006). 5′-Adenosinephosphosulphate reductase (CysH) protects *Mycobacterium tuberculosis* against free radicals during chronic infection phase in mice. *Mol. Microbiol.* 59 1744–1753 10.1111/j.1365-2958.2006.05075.x 16553880

[B70] Sherman D. R. Mdluli K. Hickey M. J. Barry C. E. III Stover C. K. (1999). AhpC, oxidative stress and drug resistance in *Mycobacterium tuberculosis. Biofactors* 10 211–217 10.1002/biof.5520100219 10609885

[B71] Sherman D. R. Sabo P. J. Hickey M. J. Arain T. M. Mahairas G. G. Yuan Y. (1995). Disparate responses to oxidative stress in saprophytic and pathogenic mycobacteria. *Proc. Natl. Acad. Sci. U.S.A.* 92 6625–6629 10.1073/pnas.92.14.6625 7604044 PMC41571

[B72] Storz G. Altuvia S. (1994). OxyR regulon. *Methods Enzymol.* 234 217–223 10.1016/0076-6879(94)34088-9 7528872

[B73] Stover C. K. de la Cruz V. F. Fuerst T. R. Burlein J. E. Benson L. A. Bennett L. T. (1991). New use of BCG for recombinant vaccines. *Nature* 351 456–460 10.1038/351456a0 1904554

[B74] Stratmann J. Strommenger B. Goethe R. Dohmann K. Gerlach G. F. Stevenson K. (2004). A 38-kilobase pathogenicity island specific for *Mycobacterium avium* subsp. *paratuberculosis* encodes cell surface proteins expressed in the host. *Infect. Immun.* 72 1265–1274 10.1128/IAI.72.3.1265-1274.2004 14977927 PMC355995

[B75] Suwandi A. Bargen I. Roy B. Pils M. C. Krey M. Zur Lage S. (2014). Experimental colitis is exacerbated by concomitant infection with *Mycobacterium avium* ssp. *paratuberculosis. Inflamm. Bowel Dis.* 20 1962–1971 10.1097/MIB.0000000000000157 25144571

[B76] Tao K. Zou C. Fujita N. Ishihama A. (1995). Mapping of the OxyR protein contact site in the C-terminal region of RNA polymerase alpha subunit. *J. Bacteriol.* 177 6740–6744. 7592462 10.1128/jb.177.23.6740-6744.1995 PMC177537

[B77] Tseng H. J. McEwan A. G. Apicella M. A. Jennings M. P. (2003). OxyR acts as a repressor of catalase expression in *Neisseria gonorrhoeae*. *Infect. Immun.* 71 550–556 10.1128/IAI.71.1.550-556.2003 PMC143252 12496210

[B78] van Vliet A. H. Baillon M. L. Penn C. W. Ketley J. M. (1999). *Campylobacter jejuni* contains two fur homologs: characterization of iron-responsive regulation of peroxide stress defense genes by the PerR repressor. *J. Bacteriol.* 181 6371–6376. 10515927 10.1128/jb.181.20.6371-6376.1999 PMC103772

[B79] Webb J. R. Vedvick T. S. Alderson M. R. Guderian J. A. Jen S. S. Ovendale P. J. (1998). Molecular cloning, expression, and immunogenicity of MTB12, a novel low-molecular-weight antigen secreted by *Mycobacterium tuberculosis*. *Infect. Immun.* 66 4208–4214. 9712769 10.1128/iai.66.9.4208-4214.1998 PMC108507

[B80] Weigoldt M. Meens J. Bange F. C. Pich A. Gerlach G. F. Goethe R. (2013). Metabolic adaptation of *Mycobacterium avium* subsp. *paratuberculosis* to the gut environment. *Microbiology* 159 380–391 10.1099/mic.0.062737-0 23223439

[B81] Weigoldt M. Meens J. Doll K. Fritsch I. Mobius P. Goethe R. (2011). Differential proteome analysis of *Mycobacterium avium* subsp. *paratuberculosis* grown in vitro and isolated from cases of clinical Johne’s disease. *Microbiology* 157 557–565 10.1099/mic.0.044859-0 21051485

[B82] Wildermann P. J. Sowa N. A. FitzGerald D. J. FitzGerald P. C. Gottesman S. Ochnser U. A. (2004). Identification of tandem duplicate regulatory small RNAs in *Pseudomonas aeruginosa* involved in iron homeostasis. *Proc. Natl. Acad. Sci. U.S.A.* 101 9792–9797 10.1073/pnas.0403423101 15210934 PMC470753

[B83] Wilson T. deLisle G. W. Marcinkeviciene J. A. Blanchard J. S. Collins D. M. (1998). Antisense RNA to ahpC, an oxidative stress defence gene involved in isoniazid resistance, indicates that AhpC of *Mycobacterium bovis* has virulence properties. *Microbiology* 144 2687–2695 10.1099/00221287-144-10-2687 9802010

[B84] Wood Z. A. Schröder E. Harris J. R. Poole L. B. (2003). Structure, mechanism and regulation of peroxiredoxins. *Trends Biochem. Sci.* 28 32–40 10.1016/S0968-0004(02)00003-8 12517450

[B85] Wu C. W. Schmoller S. K. Shin S. J. Talaat A. M. (2007). Defining the stressome of *Mycobacterium avium* subsp. *paratuberculosis* in vitro and in naturally infected cows. *J. Bacteriol.* 189 7877–7886 10.1128/JB.00780-07 17693514 PMC2168719

[B86] Yoshikawa T. Naito Y. (2000). The role of neutrophils and inflammation in gastric mucosal injury. *Free Radic.* *Res.* 33 785–794 10.1080/10715760000301301 11237100

[B87] Zahrt T. C. Song J. Siple J. Deretic V. (2001). Mycobacterial FurA is a negative regulator of catalase-peroxidase gene katG. *Mol. Microbiol.* 39 1174–1185 10.1111/j.1365-2958.2001.02321.x 11251835

[B88] Zheng M. Doan B. Schneider T. D. Storz G. (1999). OxyR and SoxRS regulation of fur. *J. Bacteriol.* 181 4639–4643. 10419964 10.1128/jb.181.15.4639-4643.1999 PMC103597

