# Supplementary material for: FurA contributes to the oxidative stress response regulation of Mycobacterium avium ssp. paratuberculosis
Source: Front Microbiol. 2015 Feb 6;6:16. doi: 10.3389/fmicb.2015.00016 (PMC4319475; doi:10.3389/fmicb.2015.00016)
Supplement: Supplementary file 2 [file Data_Sheet_2.PDF]

Suppl. Methods

Construction of allelic exchange substrate (AES) cosmid pMFur1510

Construction of a *fur*A (*map*1669c) deletion mutant of *Mycobacterium avium* subspecies *paratuberculosis* (MAP) was performed by specialized transduction according to Park and colleagues (Park *et al.*, 2008). Two primer pairs were generated to amplify PCR-fragments flanking the *fur*A gene, oFurA1 / oFurA 2 for the upstream (1094 bp) and oFurA3 / oFurA4 for the downstream (1022 bp) element. Both PCR products were cloned into the pCR2.1® TOPO vector resulting in pMFur920 and pMFur930, respectively. The plasmids were cut with AflII/ XbaI for the up- and SpeI/ XhoI for the downstream element and ligated into the pYUB854 cosmid. The inserted fragments of the resulting cosmid pMFur1510 were controlled by DNA sequencing.

Construction of mycobacteriophage for *fur*A deletion

The allelic exchange substrate (AES) pMFur1510 was digested with PacI and ligated into the PacI-restricted concatemer of the phasmid phAE87. GIGApack® III Plus Packaging Extract (Agilent Technologies) was used to pack λ-phages and transduce *E. coli* HB101. Hygromycin resistant transformants were selected, prepared, and phasmid integrity was confirmed by PCR, restriction digestion and sequencing (data not shown).

Correct cosmids were pooled and transformed into electro-competent *M. smegmatis* mc^2 155 cells. 100 µl aliquots of electroporated cells were mixed with 200 µl *M. smegmatis* mc^2 155, grown to an OD_{600} of 1.0, and 1.5 ml MB top agarose. These reactions were incubated on MBP agar plates for 5 days at 30°C and plaques were picked with sterile Pasteur pipettes and kept in MB buffer at 4°C overnight. 5 µl of each phage solution were mixed with 200 µl *M. smegmatis* mc^2 155 and 5 ml MB top agarose as described above, platted on two MBP plates and incubate in parallel at 42°C and 30°C, respectively, to confirm the temperature-sensitivity. One temperature sensitive phage (phAE151) was controlled for integrity of the AES by sequencing of the corresponding PCR fragments.

Phage Infection of *M. smegmatis,* titration of mycobacteriophage, confirmation of temperature-sensitive mycobacteriophage phenotype and the preparation of high titer mycobacteriophage stocks were essentially performed as described by Braunstein *et al.*, 2002. Phage DNA was isolated in accordance to the protocol of Sambrook *et al.,* 2001.

Specialized transduction of MAP

The protocol was performed in according to Park and colleagues (Park *et al.*, 2008) with some modifications. MAP was grown to an OD_{600} to 1.0 in MB7H9 containing OADC and Tween 80®. The bacteria were singularized by vortexing with glass beads (2-3 mm diameter) for 15 min. Then, the culture was allowed to stand for 30 min on ice and the supernatant was transferred in a sterile 50 ml reaction tube. The bacteria were centrifuged at 7200 x *g* at RT for 10 min and washed three times in 30 ml MP buffer. Afterwards, the pellet was resuspended in 5 ml MP buffer and put on ice for 20 min to reduce clumps in the culture. Following, 1 ml of the supernatant was mixed with 1 ml of mycobacterial phage lysate in a 12 ml tube in a MOI of 10:1 and incubated for 4 h at 37°C. 2 ml MB broth containing 5 % OADC were added and the samples were incubated on a shaker (100 rpm) at 37°C for 24 h. Then, the culture was placed on ice for 30 min and 300 µl of the supernatant were plated on pre-warmed (37°C) MB 7H10 agar plates containing OADC and hygromycin B with concentration of 50 µg/ml and of 75 µg/ml. The plates were incubated for 8 weeks at 37°C and hygromycin resistant clones were tested for the successful *fur*A deletion by PCR, real-time PCR and Southern blot analysis.

Generation of an antiserum against MAP specific HBHA (map3968)

The gene was cloned into the expression vector pGEX-1LambdaT (GE Healthcare, Freiburg, Germany) and expressed in *E. coli*. The purified HBHA protein was used to raise antibodies in rabbits (Seqlab, Göttingen, Germany). The resulting serum was directly used for immunofluorescence in a dilution of 1:100.

Reference List / Supplementary material

Bardarov,S., Bardarov S Jr, Pavelka,M.S., Jr., Sambandamurthy,V., Larsen,M., Tufariello,J., Chan,J., Hatfull,G., and Jacobs,J.W., Jr. (2002) Specialized transduction: an efficient method for generating marked and unmarked targeted gene disruptions in *Mycobacterium tuberculosis*, *M. bovis* BCG and *M. smegmatis Microbiology* **148**: 3007-3017.

Bardarov,S., Kriakov,J., Carriere,C., Yu,S., Vaamonde,C., McAdam,R.A., Bloom,B.R., Hatfull,G.F., and Jacobs,W.R., Jr. (1997) Conditionally replicating mycobacteriophages: a system for transposon delivery to *Mycobacterium tuberculosis Proc.Natl.Acad.Sci.U.S.A* **94**: 10961-10966.

Braunstein,M., Bardarov,S.S., and Jacobs,W.R., Jr. (2002) Genetic methods for deciphering virulence determinants of *Mycobacterium tuberculosis Methods Enzymol.* **358**: 67-99.

Boyer,H.W., Roulland-Dussoix,D. (1969) A complementation analysis of the restriction and modification of DNA in *Escherichia coli J.Mol.Biol.* **41**: 459-472.

Jark,U., Ringena,I., Franz,B., Gerlach,G.F., Beyerbach,M., and Franz,B. (1997) Development of an ELISA technique for serodiagnosis of bovine paratuberculosis *Vet.Microbiol.* **57**: 189-198.

Park,K.T., Dahl,J.L., Bannantine,J.P., Barletta,R.G., Ahn,J., Allen,A.J., Hamilton,M.J., and Davis,W.C. (2008) Demonstration of allelic exchange in the slow-growing bacterium *Mycobacterium avium* subsp. *paratuberculosis*, and generation of mutants with deletions at the pknG, relA, and lsr2 loci *Appl.Environ.Microbiol.* **74**: 1687-1695.

Raleigh, F.A., Lech, K., Brent, R., 1989. Select topics from classical bacterial genetics. In: Ausubel, F.M., et al. (Eds.), Current Protocols in Molecular Biology. Publishing Associates and Wiley Interscience, NY, USA.

Sambrook,J., Russell,D.W. (2001) *Molecular Cloning: a Laboratory Manual, 3 nd edn.* Cold Sping Harbor, N.Y.: Cold Spring Harbor Laboratory.

Snapper,S.B., Melton,R.E., Mustafa,S., Kieser,T., and Jacobs,W.R., Jr. (1990) Isolation and characterization of efficient plasmid transformation mutants of *Mycobacterium smegmatis Mol.Microbiol.* **4**: 1911-1919.
